# Neutrophil-to-lymphocyte ratio predicts inpatient gout recurrence: a large-scale multicenter retrospective cohort with machine-learning validation

**DOI:** 10.3389/fimmu.2025.1688516

**Published:** 2025-11-10

**Authors:** Hui Zhang, Jiani Liu, Ruifeng Lin, Danning Xie, Wenxia Lin, Qingqing Zhang, Fei Zhong, Shixian Chen, Qin Huang, Min Zhang, Yixin Chen, Xiaoling Chen, Zhipeng Cheng, Jiabao Xu, Li Cai, Xinhao Xia, Yaqi Chen, Ziwen Xu, Yi Yuan, Meng Li, Juan Li

**Affiliations:** 1 Department of Rheumatology and Immunology, Nanfang Hospital, Southern Medical University, Guangzhou, Guangdong, China; 2 Department of Traditional Chinese Internal Medicine, School of Traditional Chinese Medicine, Southern Medical University, Guangzhou, Guangdong, China; 3 The First School of Clinical Medicine, Southern Medical University, Guangzhou, Guangdong, China; 4 Medical College, Hunan University of Arts and Science, Changde, Hunan, China; 5 School of Integrated Traditional Chinese and Western Medicine, Guangzhou Medical University, Guangzhou, Guangdong, China; 6 Department of Rheumatology, Southern Medical University Hospital of Integrated Traditional Chinese and Western Medicine, Southern Medical University, Guangzhou, Guangdong, China

**Keywords:** neutrophil-to-lymphocyte ratio, gout, recurrence, machine learning, model

## Abstract

**Background:**

The neutrophil-to-lymphocyte ratio (NLR) is an accessible marker of systemic inflammation. However, its prognostic value for inpatient gout recurrence, particularly in comparison with traditional biomarkers, remains unclear. This study aims to investigate the association of NLR with inpatient gout recurrence, and compare its performance with traditional markers.

**Methods:**

In this international, multicenter retrospective cohort study, hospitalized patients with gout were enrolled from the GoutRe cohort (China, 2010-2025) and MIMIC-IV cohort (USA, 2008-2019). Restricted cubic spline, Cox regression and competing risk models were deployed to visualize and assess the association of NLR with inpatient gout recurrence risk. Model performance was evaluated using the C-statistic, net reclassification improvement, and decision curve analysis. Multiple machine learning algorithms were employed for external validation.

**Results:**

Among 7,603 patients (GoutRe: 5,584; MIMIC-IV: 2,019), elevated NLR (>2.69) was independently associated with a higher inpatient gout recurrence risk (GoutRe: HR = 2.05; MIMIC-IV: HR = 2.84; both *P* < 0.001). NLR correlated with systemic inflammation, comorbidities, and use of diuretics/β-blockers. It outperformed serum uric acid (UA) and C-reactive protein (CRP) in predicting inpatient gout recurrence (AUC: 0.62 *vs.* 0.59 and 0.61, respectively), with improved accuracy when combined with UA (AUC = 0.65, *P* < 0.01). Predictive value remained consistent across subgroups, including those with normal UA, no tophus, and ongoing anti-inflammatory or urate-lowering therapy. Machine learning models, particularly XGBoost, confirmed NLR’s predictive strength. Incorporation of NLR into baseline models improved discrimination and reclassification. Decision curve analysis showed greater net clinical benefit with NLR-based models. Biological plausibility analysis revealed that elevated NLR reflected neutrophilia and lymphopenia, indicative of systemic inflammation during the intercritical period.

**Conclusions:**

Elevated NLR is a robust, accessible biomarker independently associated with inpatient gout recurrence. Its integration into clinical risk models enhances prediction accuracy and supports personalized inpatient gout recurrence prevention strategies.

## Introduction

Gout is a prevalent form of chronic inflammatory arthritis, affecting over 55.8 million individuals worldwide (1). Despite the broad availability of urate-lowering therapies (ULT), approximately 70% of patients remain inadequately controlled (2). The recurrent nature of gout, along with its systemic complications (3, 4), such as cardiovascular and renal comorbidities (5–8), imposes a substantial burden on both patients and healthcare systems. This burden is especially pronounced among hospitalized patients with comorbid gout, in whom the recurrence rate of acute flares ranges from 14% to 43% (9). Acute flares during hospitalization have many adverse effects, including acute episodes of intense joint pain and swelling, increased healthcare expenditures, joint damage, increased risk of kidney disease, cardiovascular disease, venous thromboembolism, and diminished quality of life (10, 11).

Traditionally, serum uric acid (UA) levels, tophus presence, and inflammatory markers such as C-reactive protein (CRP) have been used to monitor disease activity and guide treatment (4, 12–16). However, their utility in predicting gout recurrence, particularly in hospitalized patients with normal UA levels or those undergoing ULT (17, 18), remains limited. Notably, flares often occur despite achieving target UA levels (19), highlighting a disconnect between biochemical control and clinical outcomes. This discrepancy underscores the need for reliable, accessible biomarkers that can dynamically reflect the risk of acute inflammatory episodes and guide timely preventive strategies.

Recent advances in gout pathophysiology suggest that systemic inflammation and immune dysregulation are central to recurrence (20). The neutrophil-to-lymphocyte ratio (NLR), derived from routine complete blood counts (21, 22), is an emerging indicator of systemic inflammation that reflects the balance between innate and adaptive immunity. Previous studies have demonstrated the prognostic value of NLR in various autoimmune and inflammatory conditions, including rheumatoid arthritis, systemic lupus erythematosus, and ankylosing spondylitis. However, to our knowledge, no study has systematically evaluated the role of NLR in predicting inpatient gout recurrence or compared its performance with conventional biomarkers in hospitalized patients (14, 23, 24).

To address this knowledge gap, we conducted a multicenter retrospective cohort study using data from the GoutRe cohort in China and the MIMIC-IV cohort in the United States. We aimed to (1) investigate the independent association between NLR and inpatient gout recurrence, (2) identify an optimal NLR threshold for risk stratification, and (3) evaluate the incremental predictive value of NLR over conventional markers such as UA and CRP using both statistical and machine learning approaches. Our findings may establish NLR as a cost-effective, accessible, and clinically actionable biomarker for predicting inpatient gout recurrence.

## Methods

### Study design and population: a multicenter retrospective study

This retrospective, multicenter study involved two cohorts: the Gout Recurrence (GoutRe) multicenter cohort, consisting of patients from five tertiary hospitals in China (Nanfang Hospital, Ganzhou People’s Hospital, Southern Medical University Hospital of Integrated Traditional Chinese and Western Medicine, Taishan People’s Hospital, and Dongguan Hospital of Traditional Chinese Medicine), and the Medical Information Mart for Intensive Care IV (MIMIC-IV) cohort, which includes de-identified health-related data from hospital admissions at the Beth Israel Deaconess Medical Center in Boston, USA. In this study, we focused on non-ICU hospitalized patients to minimize potential bias from critical illness on outcome assessment. The GoutRe cohort included patients hospitalized between January 1, 2010 and May 1, 2025, while the MIMIC-IV cohort used data from 2008 to 2019. The inclusion of diverse populations enhanced the generalizability and robustness of our findings. The GoutRe cohort was established to investigate recurrence risk and predictors inpatient gout recurrence in real-world clinical settings. It has been described in our previous study (25).

**Figure 1:**
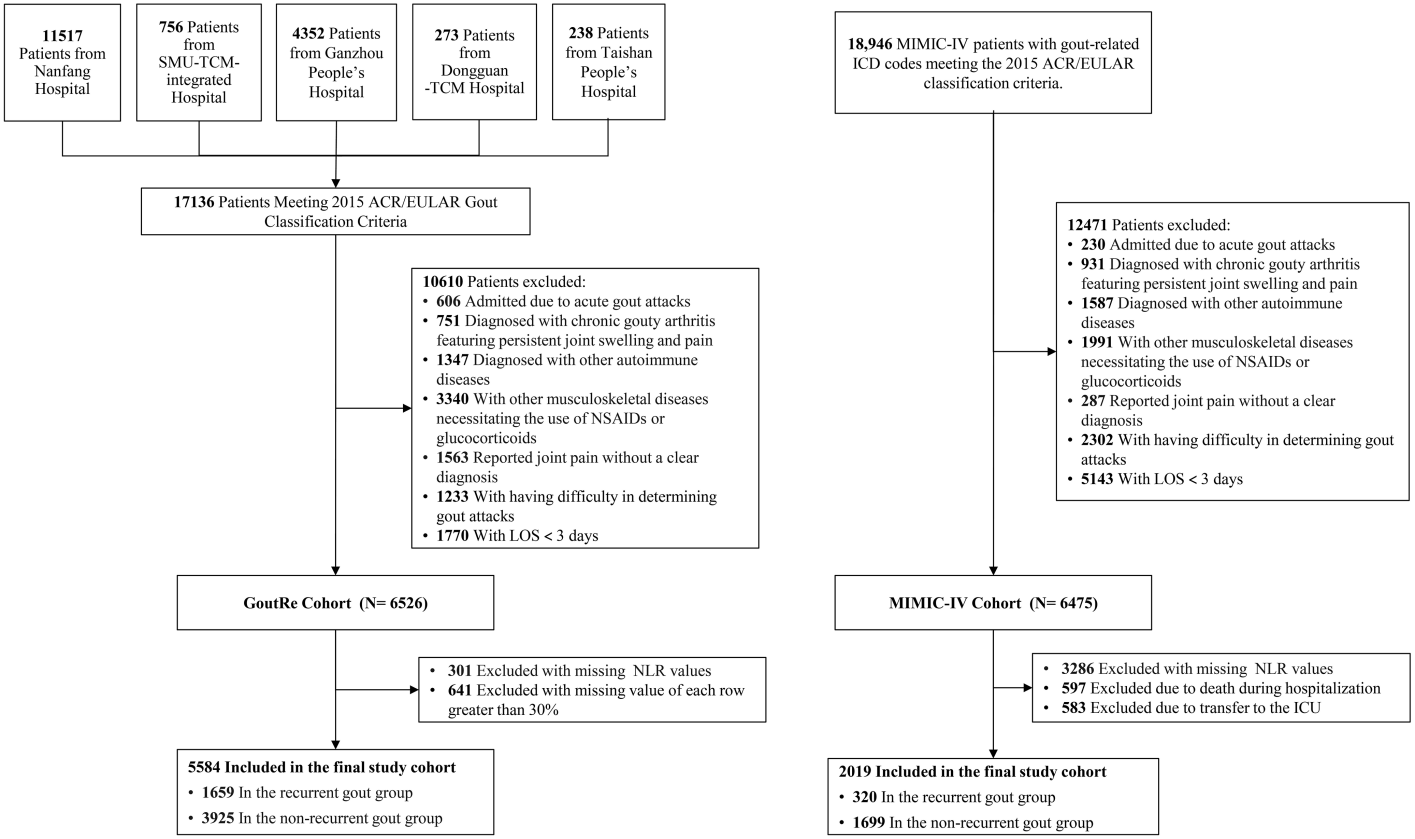
The flowchart of the GoutRe cohort and the MIMIC-IV cohort. SMU, Southern Medical University; TCM, Traditional Chinese Medicine; NSAIDs, Nonsteroidal Anti-inflammatory Drugs; LOS, Length of Stay; NLR, Neutrophil-Lymphocyte Ratio; ICD, International Classification of Diseases; ICU, intensive care unit.

A total of 36,082 patients (GoutRe cohort: 17,136; MIMIC-IV cohort: 18,946) across both cohorts met the 2015 ACR/EULAR gout classification criteria and had a gout-related ICD-10 code in their discharge diagnosis. Exclusion criteria included admission due to acute gout attacks, chronic gouty arthritis, autoimmune diseases (including lupus erythematosus, Sjögren’s syndrome, systemic sclerosis, dermatomyositis, antiphospholipid antibody syndrome, polyarteritis nodosa, Wegener’s granulomatosis, giant cell arteritis, rheumatoid vasculitis, Behçet’s syndrome, and other connective tissue disorders), and musculoskeletal diseases necessitating the use of NSAIDs or glucocorticoids (including rheumatoid arthritis, psoriatic arthritis, ankylosing spondylitis, osteoarthritis, polymyalgia rheumatica, and other inflammatory, infectious, or degenerative joint disorders). Individuals with enthesopathies or a personal or family history of arthritis were also excluded. The specific diseases and corresponding ICD-10 codes used for both inclusion and exclusion criteria are listed in **Supplementary Table S4**. Reported joint pain without a clear diagnosis, difficulty in determining gout attacks, and a length of stay (LOS) of fewer than 3 days were also exclusion criteria (**Figure 1**). After applying the exclusion criteria, 6,526 patients were initially included in the GoutRe cohort and 6,475 patients in the MIMIC-IV cohort.

Patients with missing NLR values (301 in the GoutRe cohort and 3,286 in the MIMIC-IV cohort) or missing data exceeding 30% (641 in the GoutRe cohort) were further excluded. Additionally, patients who died during hospitalization (597 in the MIMIC-IV cohort) or were transferred to the ICU (583 in the MIMIC-IV cohort) were excluded, resulting in a final study population of 5,584 patients in the GoutRe cohort and 2,019 patients in the MIMIC-IV cohort (**Figure 1**) (26).

This study was approved by the institutional ethics committees of all participating centers (NFEC-2023-562, NFEC-2023-577, TY-ZKY2024-081-01, 202409-K3-0, PJ[2025]73), and all procedures complied with the Declaration of Helsinki (1975, as revised in 2013). Written informed consent was waived due to the retrospective design. Access to the MIMIC-IV database was approved (Record ID: 63866361). Detailed cohort characteristics, inclusion and exclusion criteria, and data-processing workflows are described in the **Supplementary Methods**.

### Data collection and assessment of inpatient gout recurrence

The primary outcome was the occurrence of inpatient gout recurrence, defined as a new acute gout flare in patients with a prior history of gout. Inpatient gout recurrence was diagnosed in patients admitted for conditions other than gout, based on the appearance of symptoms of a gout flare during hospitalization and the recorded administration of anti-gout medications. Previous studies have shown high accuracy in identifying primary emergency department visits and hospitalizations for gout using ICD-10 code (21, 26–30). Inpatient gout recurrence was evaluated by the attending physician or a senior clinician, with only the first flare episode during admission being analyzed for patients experiencing multiple episodes. Patients were categorized into two groups based on whether they experienced inpatient gout recurrence or not.

First, we identified all admissions that received the discharge comorbid diagnosis of Gout according to ICD-10. Comorbid diagnoses were recorded separately from the primary admission diagnosis. Second, a structured query language “word search” was conducted to search for specific words which could appear anywhere within the electronic discharge letters. Search terms included “gout”, “allopurinol”, “febuxostat”, and “colchicine”. The search yielded a list of admissions containing at least one of the words of interest. Data were collected from physical hospital records and electronic laboratory databases.

### Measurement of NLR

NLR was calculated by dividing the absolute neutrophil count by the absolute lymphocyte count (31). In the GoutRe cohort, baseline NLR was determined on the first day of admission, prior to any treatment initiation. Neutrophil and lymphocyte counts were obtained through a complete blood count analysis of blood specimens and reported as ×10^9^ cells/µL. While the neutrophil and lymphocyte counts for patients with recurrent gout in the MIMIC-IV database were extracted from the earliest available records after admission, confirming that the data were collected prior to the onset of the gout attack.

### Covariates

Covariates included demographic characteristics, lifestyle factors, laboratory tests, physical examination, comorbidities, and medication usage. Demographic characteristics comprised age, sex, race, and weight changes. Lifestyle factors included smoking history and alcohol consumption history. Laboratory tests encompassed complete blood count parameters and kidney function tests. Physical examination findings included the presence of tophus. Comorbidities assessed were hypertension, diabetes mellitus, cardiovascular disease, heart failure, stroke, dyslipidemia, fatty liver disease, renal disease, thyroid disorders, cancer, history of nephrolithiasis, and metabolic syndrome. Medication usage included anti-gout medications, hypoglycemic agents, cardiovascular drugs, anticoagulants, lipid-modifying agents, and mannitol. Additional details were provided in the **Supplementary Materials** (**Supplementary Tables S2**-**S5**; **Supplementary Methods**).

### Statistical analysis

The normality of continuous variables was assessed using the Shapiro-Wilk test. Continuous variables were compared between different NLR groups using independent t-tests or non-parametric tests when necessary. Categorical variables were analyzed using chi-square or Fisher’s exact tests. Missing data were addressed using Multiple Imputation by Chained Equations with the classification and regression trees method, and the results from each imputed dataset were pooled to obtain final estimates. The optimal NLR cutoff value was determined by maximizing the Gray’s test statistic in the competing risks framework (31) and the log-rank test statistic in the Cox proportional hazards model. Cox regression analysis was performed with the time scale defined as the time from admission (start time) to the first recorded gout recurrence during hospitalization (event time), and the follow-up time was censored at discharge (end time). Since discharge may impact the outcome event, we treated discharge as a competing event and applied a competing risk model to assess the recurrence risk. Sensitivity analyses were conducted using both competing risk models and Kaplan-Meier survival analysis to confirm the robustness of the findings across different model specifications.

To estimate the association between NLR and inpatient gout recurrence, Cox proportional hazards regression models were used to compute hazard ratios (HRs), 95% confidence intervals (CIs), and P-values. Subgroup analyses explored the consistency of NLR effects across age, sex, and comorbidities. Interaction P-values were calculated using likelihood ratio tests, comparing models with and without interaction terms for each stratification factor. To further assess predictive performance, machine learning models were employed alongside traditional Cox regression models. These included random survival forests, support vector machines (SVM), and Extreme Gradient Boosting (XGBoost) models. Predictive accuracy was assessed by generating ROC curves and calculating the area under the curve (AUC) for each model. The DeLong test (32) was used to compare the statistical significance of differences between the AUCs. Additionally, Net Reclassification Improvement (NRI) (33) and Integrated Discrimination Improvement (IDI) (34) indices were computed to assess the incremental predictive value of incorporating NLR into the baseline models, with confidence intervals obtained via bootstrap resampling. Decision curve analysis (DCA) was conducted to evaluate the clinical utility of NLR-based models across a range of threshold probabilities, assessing the net benefit of various prediction models in clinical context.

All statistical tests were two-sided, and P values less than 0.05 were considered statistically significant. All analyses were performed using R version 4.3.2 (R Foundation for Statistical Computing). Machine-learning models were implemented using the randomForestSRC, e1071, and xgboost packages for Random Survival Forest (RSF), Support Vector Machine (SVM), and Extreme Gradient Boosting (XGBoost), respectively. Other statistical procedures—including Cox regression, Fine-Gray competing-risk modeling, multiple imputation, ROC/AUC analysis and DeLong testing, NRI/IDI, and decision curve analysis—were performed using the survival, riskRegression, mice, pROC, survIDINRI, and rmda packages. Package versions and key hyperparameter settings, as well as model evaluation procedures, are provided in the **Supplementary Materials** to ensure transparency and reproducibility.

## Results

### Baseline characteristics

A total of 7,603 participants were eligible for the current study, comprising 5,584 patients from the GoutRe cohort (100% Asian; mean [SD] age, 62.7 [14.7] years; 86.3% male) and 2,019 patients from the MIMIC-IV cohort (72.9% White; mean [SD] age, 68.0 [12.9] years; 75.1% male) (**Supplementary Table S13**, **Supplementary Figure S1**). The GoutRe cohort consisted of 1,659 patients with inpatient gout recurrence and 3,925 without recurrence, while the MIMIC-IV cohort included 320 and 1,699 patients in the recurrent and non-recurrent groups, respectively.

**Figure 2:**
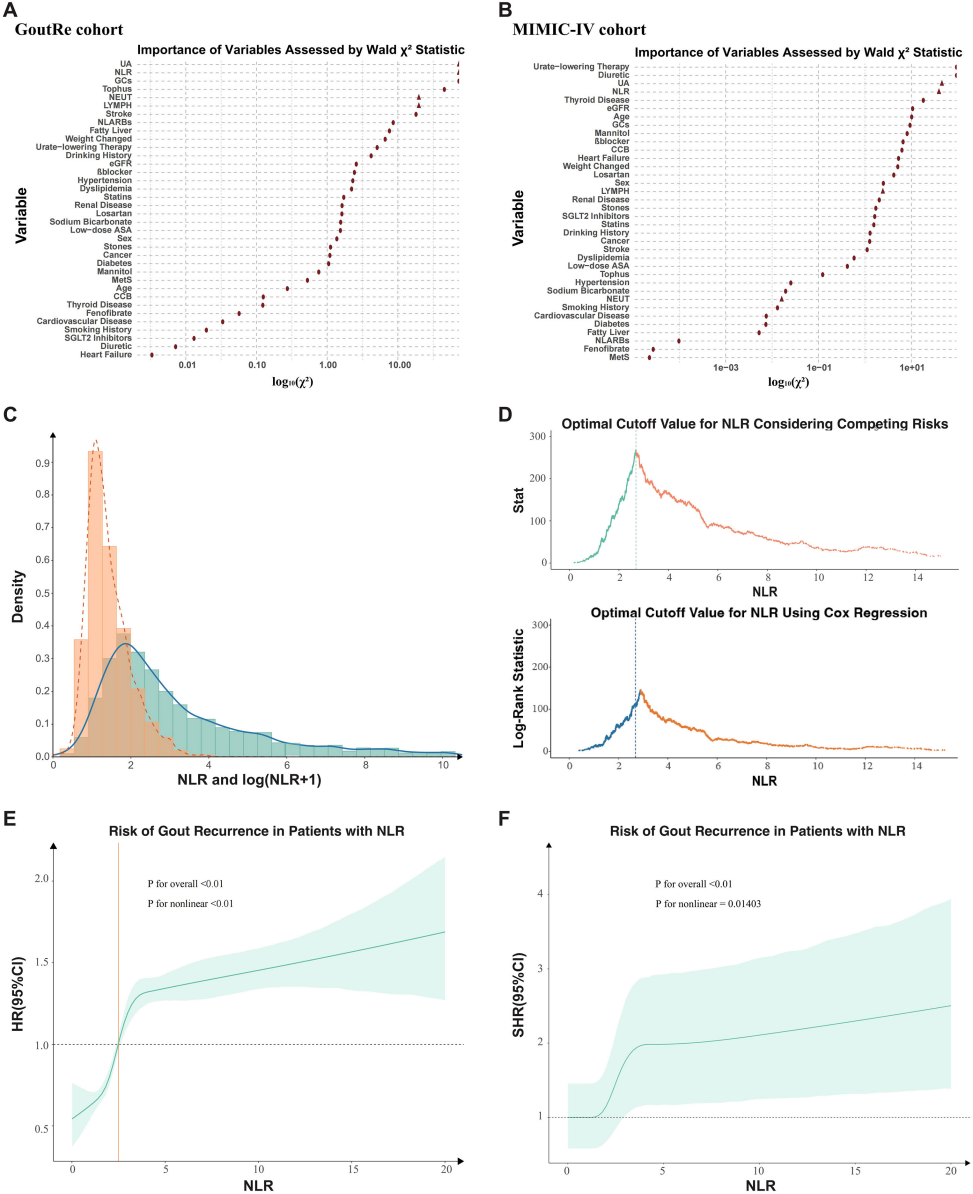
Distribution and predictive value of NLR in determining optimal cutoff and risk of inpatient gout recurrence. **(A, B)** Importance of Each Variable in the Full Model as Measured by log10(Partial Wald χ² Minus the Predictor Degrees of Freedom). **(C)** The distribution of neutrophil-to-lymphocyte ratio (NLR). **(D)** The optimal cutoff value of 2.69 was determined by maximizing the double statistics of the competing risk model and Cox proportional hazards model. **(E)** Association Between NLR and inpatient Gout Recurrence Risk (Cox Proportional Hazards Model). The solid line showed the hazard ratio (HR) for inpatient gout recurrence across NLR levels, with the shaded area representing the 95% confidence interval. Overall and nonlinear P valued indicate the significance of NLR and its non-linearity. **(F)** Association Between NLR and inpatient Gout Recurrence Risk (Competing Risks Model). The solid line represented the sub-distribution hazard ratio (SHR) for inpatient gout recurrence across NLR levels, with the shaded area indicating the 95% confidence interval. Overall and nonlinear P values assessed the significance and nonlinearity of NLR. NLR, Neutrophil-Lymphocyte Ratio; β-blocker, Beta-blocker; eGFR, Estimated glomerular filtration rate; GCs, Glucocorticoids; CCB, Calcium channel blockers; UA, Uric Acid; ASA, Acetylsalicylic acid; NL-ARBs, Non-losartan angiotensin II receptor blockers; MetS, Metabolic Syndrome.

Participants were stratified by NLR based on an optimal cutoff value of **2.69**, determined by maximizing the log-rank statistic within a Cox proportional hazards model (**Figure 2C**). In the GoutRe cohort, patients in the higher NLR group (n = 2,736) were older (mean age, 65.0 [14.4] years vs 60.5 [14.6] years, *P* < 0.01) and had higher rates of inpatient gout recurrence (39.69% vs 20.12%, *P* < 0.01), tophus (7.97% vs 3.83%, *P* < 0.01), nephrolithiasis (44.0% vs 40.5%, *P* < 0.01), and heart failure (8.52% vs 3.65%, *P* < 0.01) compared to the lower NLR group (n = 2,848). They also exhibited higher UA levels, lower eGFR levels, and greater use of diuretics (24.49% vs 9.13%, *P* < 0.01) and β-blockers (27.96% vs 18.57%, *P* < 0.01).

Similarly, in the MIMIC-IV cohort, the higher NLR group (n = 1,437) had a higher inpatient gout recurrence rate (18.16% vs 10.14%, *P* < 0.01), heart failure prevalence, and more frequent use of diuretics and β-blockers compared to the lower NLR group (n = 582). A comprehensive summary of baseline characteristics and group differences is provided in **Supplementary Table S13**.

### Association of NLR with inpatient gout recurrence

The distribution of NLR in the GoutRe cohort is illustrated in **Figure 2C**, where the majority of values clustered between 2 and 3. A nonlinear relationship between NLR and inpatient gout recurrence was identified (**Figures 2E, F**), with *P* values for nonlinearity being highly significant (*P* < 0.001). The optimal NLR cutoff value of **2.69** was determined using a combination of log-rank test maximization within the survival analysis framework and Gray’s test within the competing risk model framework (**Figure 2D**). The predictive importance of NLR was comparable to predictors including UA and CRP (**Figure 2A**), a finding that was validated in the MIMIC-IV cohort, further supporting the robustness of NLR as a significant predictor of inpatient gout recurrence (**Figure 2B**).

**Figure 3:**
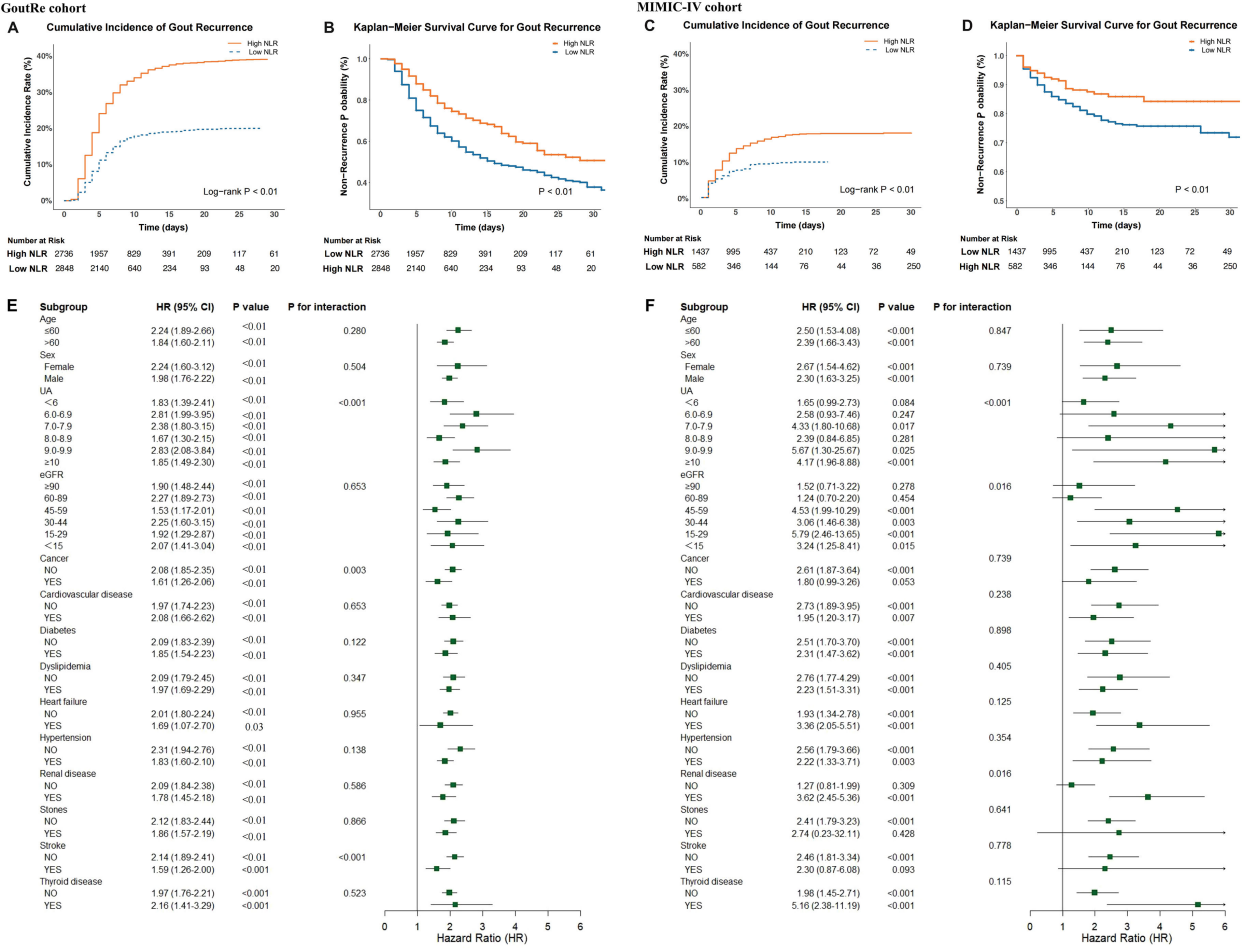
Association between NLR and inpatient gout recurrence in the GoutRe and MIMIC-IV cohorts. **(A, C)** Cumulative incidence function plots illustrating the probability of inpatient gout recurrence, stratified by high *vs.* low NLR in the GoutRe cohort **(A)** and the MIMIC-IV cohort **(C)**. **(B, D)** Kaplan-Meier survival curves illustrating the probability of remaining recurrence-free over time in the GoutRe cohort **(B)** and the MIMIC-IV cohort **(D)**. **(E, F)** Forest plots showing subgroup analyses of the association between elevated NLR and the risk of inpatient gout recurrence in the GoutRe cohort **(E)** and the MIMIC-IV cohort **(F)**. HRs and 95% CIs were derived from multivariable Cox proportional hazards models adjusted for demographics, comorbidities, medications, laboratory parameters, and urate-lowering therapy. *P* for interaction values indicate the heterogeneity of effect across subgroups. HR, hazard ratio; CI, confidence interval; NLR, neutrophil-to-lymphocyte ratio; UA, uric acid; eGFR, estimated glomerular filtration rate.

The cumulative incidence of inpatient gout recurrence is depicted in **Figure 3**. Patients with elevated NLR exhibited a significantly higher inpatient gout recurrence risk in both the GoutRe and MIMIC-IV cohorts (log-rank *P* < 0.001). This association was consistent across all examined subgroups, including those with normal UA levels, those without tophus, those with both normal UA levels and absence of tophus, those undergoing ULT and anti-inflammatory treatment (**Supplementary Figures S3**-**S6**). Kaplan-Meier survival curves (**Figure 3**, **Supplementary Figure S3**) further supported these observations, indicating a robust association between higher NLR and increased inpatient gout recurrence across various clinical settings. In multivariate Cox models, elevated NLR was independently associated with increased inpatient gout recurrence risk (GoutRe: HR, 2.05 [95% CI, 1.83-2.30]; *P* < 0.01; MIMIC-IV: HR, 2.84 [95% CI, 2.08-3.87]; *P* < 0.01), with specific values detailed in **Supplementary Table S8**.

### Comparative predictive value of NLR, UA, and CRP

Linear regression analysis demonstrated a fair positive association between NLR and CRP (R²= 0.091, *P* < 0.001) (**Supplementary Figure S2B**). In contrast, no significant correlation was observed between NLR and UA (R²= 8.9e-05, *P* = 0.61) (**Supplementary Figure S2C**), or between CRP and UA (R²= 0.0017, *P* = 0.026) (**Supplementary Figure S2A**). ROC curves were performed to compare the predictive ability of NLR, UA, and CRP for inpatient gout recurrence (**Supplementary Figure S2D**). The AUC for NLR was 0.62, indicating better predictive performance compared with UA (AUC = 0.59) and CRP (AUC = 0.61). The DeLong test confirmed that NLR’s predictive performance was significantly superior to UA (*P* = 0.025), while no significant difference was found between CRP and NLR (*P* = 0.623). Notably, combining NLR with UA further improved predictive performance, yielding an AUC of 0.65, which was statistically superior to any single biomarker (*P* < 0.01 for all pairwise comparisons).

### Robustness across subgroups

Stratified analyses of the GoutRe and MIMIC-IV cohorts based on age, sex, eGFR, UA levels, and comorbidities were presented in **Figure 3**. Higher NLR was consistently associated with an increased risk of inpatient gout recurrence across all subgroups. For instance, in the GoutRe cohort, the HR for patients with UA levels between 7 and 7.9 mg/dL was 2.38 (95% CI, 1.80-3.15; *P* < 0.001), while for patients with UA levels between 9 and 9.9 mg/dL, the HR was 2.83 (95% CI, 2.08-3.84; *P* < 0.001).

In both the GoutRe and MIMIC-IV cohorts, higher NLR consistently remained a strong predictor of inpatient gout recurrence across a wide range of clinical settings and comorbidities, even after adjusting for all covariates. These trends were consistent across subgroups including cancer, cardiovascular disease, diabetes, dyslipidemia, hypertension, heart failure, renal disease, stroke, thyroid disease, and nephrolithiasis, as well as across various age, sex, eGFR, and UA levels groups, with no significant interaction observed between these characteristics and NLR (*P* for interaction > 0.05). Notably, in the MIMIC-IV cohort, the HR for inpatient gout recurrence in patients with thyroid disease was 5.16 (95% CI, 2.38-11.19; *P* < 0.001), further emphasizing the strong predictive value of NLR in certain comorbid populations (**Figure 3**). Similar trends were observed in other subgroups, as detailed in **Table 1** and **Supplementary Tables S8**-**S12**.

**Table 1:** Multivariate-adjusted hazard ratios (95% CI) of NLR for inpatient gout recurrence.

Characteristic		Model 1^a^		Model 2^b^		Model 3^c^	
		HR (95% CI)	*P* value	HR (95% CI)	*P* value	HR (95% CI)	*P* value
GoutRe cohort							
NLR^d^							
	Lower	Ref	Ref	Ref	Ref	Ref	Ref
	Higher	1.86 (1.68, 2.06)	<0.01	1.95 (1.74, 2.18)	0.00	2.05 (1.83, 2.30)	<0.01
UA^e^							
	<6	Ref	Ref	Ref	Ref	Ref	Ref
	6.0-6.9	1.23 (1.02, 1.49)	0.03	1.24 (1.03, 1.50)	0.02	1.24 (1.03, 1.50)	0.03
	7.0-7.9	1.46 (1.23, 1.74)	<0.01	1.48 (1.24, 1.76)	<0.01	1.46 (1.22, 1.74)	<0.01
	8.0-8.9	1.58 (1.33, 1.87)	<0.01	1.59 (1.34, 1.88)	<0.01	1.57 (1.33, 1.87)	<0.01
	9.0-9.9	1.79 (1.50, 2.13)	<0.01	1.75 (1.47, 2.10)	<0.01	1.79 (1.49, 2.14)	<0.01
	≥10	2.32 (1.98, 2.70)	<0.01	2.29 (1.96, 2.68)	<0.01	2.27 (1.93, 2.67)	<0.01
eGFR							
	≥90	Ref	Ref	Ref	Ref	Ref	Ref
	60-89	1.12 (0.97, 1.29)	0.13	1.07 (0.92, 1.23)	0.38	1.06 (0.91, 1.24)	0.45
	45-59	1.31 (1.10, 1.55)	<0.01	1.17 (1.00, 1.39)	0.07	1.15 (0.96, 1.40)	0.13
	30-44	1.37 (1.14, 1.63)	<0.01	1.15 (0.96, 1.38)	0.13	1.12 (0.91, 1.38)	0.29
	15-29	1.34 (1.10, 1.62)	<0.01	1.05 (0.86, 1.28)	0.64	1.05 (0.82, 1.34)	0.70
	<15	1.21 (1.02, 1.44)	<0.01	1.03 (0.86, 1.23)	0.76	1.02 (0.80, 1.30)	0.86
Tophus							
	No	Ref	Ref	Ref	Ref	Ref	Ref
	Yes	1.98 (1.70, 2.31)	<0.01	1.89 (1.61, 2.21)	<0.01	1.81 (1.55, 2.13)	<0.01
MIMIC-IV Cohort							
NLR^d^							
	Lower	Ref	Ref	Ref	Ref	Ref	Ref
	Higher	1.71 (1.29, 2.27)	<0.01	2.12 (1.56, 2.87)	<0.01	2.84 (2.08, 3.87)	<0.01
UA^e^							
	<6	Ref	Ref	Ref	Ref	Ref	Ref
	6.0-6.9	0.84 (0.44, 1.60)	0.59	0.85 (0.44,1.64)	0.63	0.93 (0.47, 1.83)	0.83
	7.0-7.9	1.85 (1.04, 3.29)	0.04	1.85 (1.04, 3.29)	0.04	1.51 (0.82, 2.77)	0.18
	8.0-8.9	1.11 (0.56, 2.20)	0.76	1.11 (0.56, 2.20)	0.76	0.97 (0.48, 1.98)	0.91
	9.0-9.9	1.23 (0.58, 2.62)	0.59	1.23 (0.58, 2.62)	0.59	0.92 (0.41, 2.08)	0.84
	≥10	2.32 (1.39, 3.87)	<0.01	2.32 (1.39, 3.87)	<0.01	1.86 (1.05, 3.32)	0.04
eGFR							
	≥90	Ref	Ref	Ref	Ref	Ref	Ref
	60-89	0.80 (0.54, 1.16)	0.24	0.74 (0.51, 1.09)	0.13	0.92 (0.61, 1.37)	0.67
	45-59	1.21 (0.79, 1.85)	0.38	1.05 (0.68, 1.61)	0.79	2.01 (1.23, 3.27)	<0.01
	30-44	1.54 (1.05, 2.26)	0.04	1.24 (0.84, 1.84)	0.29	2.61 (1.59, 4.29)	<0.01
	15-29	1.42 (0.96, 2.10)	0.08	1.06 (0.70, 1.59)	0.75	2.30 (1.36, 3.89)	<0.01
	<15	1.32 (0.85, 2.05)	0.23	1.03 (0.65, 1.63)	0.80	1.73 (0.99, 3.04)	0.06
Tophus							
	No	Ref	Ref	Ref	Ref	Ref	Ref
	Yes	2.09 (1.04, 4.22)	0.04	1.92 (0.95, 3.88)	0.07	1.39 (0.67, 2.89)	0.38

HR, Hazard Ratio; NLR, Neutrophil-Lymphocyte Ratio; UA, Uric Acid; eGFR, Estimated glomerular filtration rate.

a Model 1 is the unadjusted model.

b Model 2 is adjusted for only the remaining three factors of NLR, UA, eGFR, and Tophus, in addition to the factors being analyzed. For example, UA, eGFR, and Tophus are adjusted when NLR is analyzed.

c Model 3 adjusted for model 2 covariates plus multivariable, including age, sex, smoking history, drinking history, weight change, hypertension, diabetes, cardiovascular disease, heart failure, stroke, dyslipidemia, fatty liver, renal disease, thyroid disease, cancer, stones, MetS, urate-lowering therapy, sodium bicarbonate, GCs, SGLT2 inhibitors, CCB, losartan, NL-ARBs, β-blockers, diuretic, low-dose aspirin, statins, fenofibrate, mannitol.

d Per 1SD of Neutrophil-Lymphocyte Ratio.

e Per 1mg/dL of uric acid.

### Incremental value of NLR

**Figure 4:**
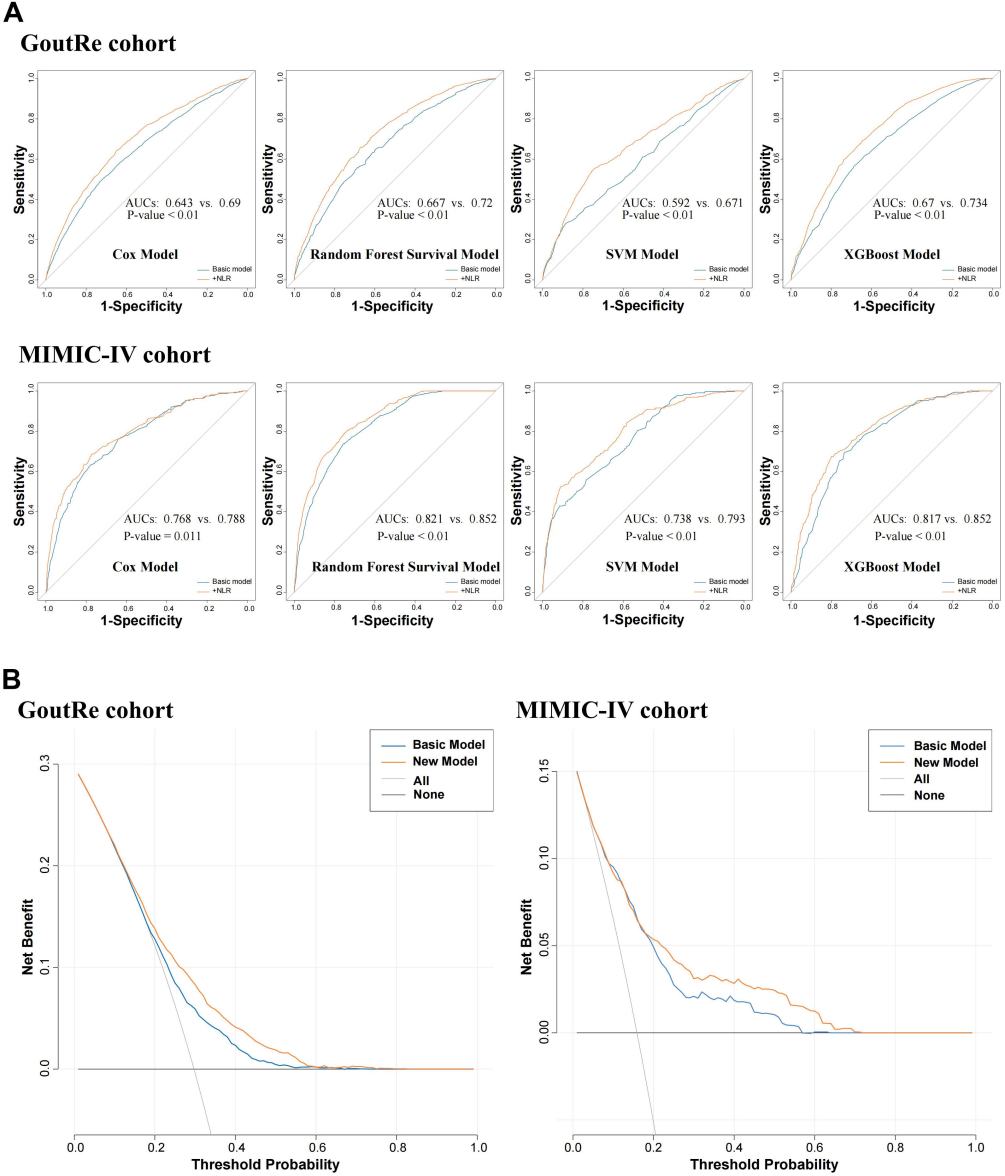
Incremental predicted value and clinical practicability evaluation analysis. **(A)** ROC curve analysis for predicting inpatient gout recurrence after incorporating NLR into the model. **(B)** Decision curve analysis for predicting inpatient gout recurrence after incorporating NLR into the model.

The addition of NLR to the baseline clinical prediction model significantly improved the accuracy of inpatient gout recurrence risk estimation, with the C-statistic increasing from 0.65 to 0.68 in the GoutRe cohort (*P* < 0.001) and from 0.80 to 0.81 in the MIMIC-IV cohort (*P* = 0.003). These improvements were further supported by significant enhancements in the IDI indices (**Supplementary Table S14**). ROC curves (**Figure 4A**) demonstrated the improved sensitivity and specificity with the inclusion of NLR. Furthermore, DCA confirmed the clinical utility of the enhanced model, showing consistent net benefit across a range of threshold probabilities in both cohorts (**Supplementary Figure S7**).

To evaluate the robustness of NLR’s predictive value across modeling strategies, four distinct approaches were applied: Cox proportional hazards regression, support vector machine (SVM), random survival forest, and XGBoost. The inclusion of the NLR significantly improved the AUC for all models across both the GoutRe and MIMIC-IV cohorts. Specifically, the XGBoost model achieved the highest AUC (GoutRe: 0.73; MIMIC-IV: 0.85), followed closely by the random survival forest model (GoutRe: 0.72; MIMIC-IV: 0.85), highlighting the strong performance and stability of tree-based ensemble models in inpatient gout recurrence risk prediction when NLR is included (**Figure 4**).

### Biological plausibility

**Figure 5:**
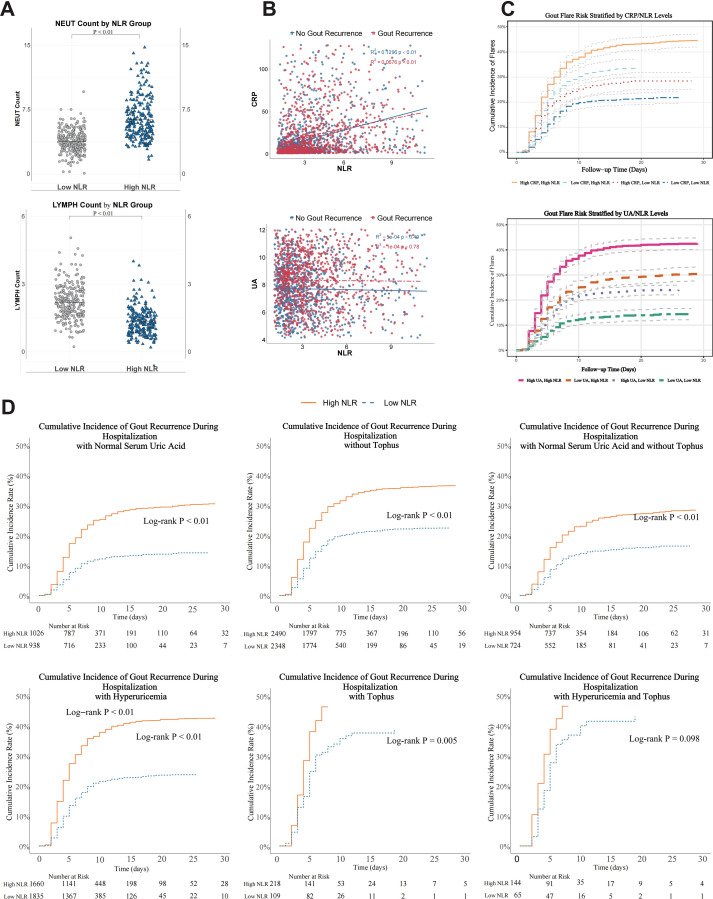
Biological plausibility analyses of the association between NLR and inpatient gout recurrence. **(A)** Absolute neutrophil and lymphocyte counts stratified by NLR groups. **(B)** Correlation between NLR and CRP levels. **(C)** inpatient gout recurrence rates stratified by combined NLR and CRP levels. **(D)** Kaplan-Meier survival curves for inpatient gout recurrence in patients with normouricemia and absence of tophus, stratified by NLR level. *P* < 0.05 was considered statistically significant.

Patients with elevated NLR exhibited significantly higher absolute neutrophil counts and lower lymphocyte counts compared to those with low NLR (both *P* < 0.01; **Figure 5A**). A moderate positive correlation was observed between NLR and CRP levels (R²= 0.13, *P* < 0.01; **Figure 5B**).

Risk stratification showed that patients with both elevated NLR and CRP had the highest inpatient gout recurrence rates, followed by those with elevated NLR alone. In contrast, patients with low NLR had lower inpatient gout recurrence risk regardless of CRP status (*P* < 0.01 for all comparisons; **Figure 5C**). The association between elevated NLR and inpatient gout recurrence remained statistically significant in patients with normal UA levels and no presence of tophus (log-rank *P* < 0.01; **Figure 5D**).

## Discussion

Given the lack of reliable predictive indicators for inpatient gout recurrence, our study addresses this gap by systematically evaluating the value of the NLR in hospitalized patients with gout. To our knowledge, this is the first study to assess the association between NLR and inpatient gout recurrence risk using large-scale, multicenter data from the GoutRe cohort in China and the MIMIC-IV database in the United States. Our results demonstrate that higher NLR levels are independently associated with an increased risk of inpatient gout recurrence, underscoring the potential of NLR as a robust and readily available biomarker for clinical risk stratification. Moreover, we not only provide explicit validation of NLR’s significance as a novel predictor for inpatient gout recurrence but also demonstrate its wide applicability across diverse patient populations, thereby enhancing the discriminatory ability and robustness of inpatient gout recurrence prediction models.

Based on our calculated cut-off value of 2.69, we stratified patients into high and low NLR groups and found a significantly elevated inpatient gout recurrence risk in the high NLR group. This finding is consistent with prior research, indicating an elevated percentage of neutrophils and a decreased percentage of lymphocytes in the peripheral blood of patients with acute gout (23).Furthermore, the NLR is higher in patients experiencing acute gout compared to those in remission (28, 30).NLR serves as an intuitive and effective indicator to assess *in vivo* inflammatory response (22, 35). Numerous studies have demonstrated the significant value of NLR in disease assessment, prediction, and evaluation of disease activity and treatment efficacy in patients with systemic lupus erythematosus and rheumatoid arthritis complicated by lupus nephritis (21, 36). Gout is an inflammatory disease, and its inpatient gout recurrence is closely associated with the level of systemic inflammation (30). During gout attacks, neutrophils play a pivotal role by migrating to the affected joint upon deposition of urate crystals and endeavoring to eliminate these crystals through phagocytosis (37). This process induces local inflammation, resulting in joint swelling and pain. Lymphocytes play a crucial role in immune system regulation, and their decrease may indicate impaired immune regulatory function and ineffective suppression of inflammation, thereby augmenting the risk of inpatient gout recurrence. Inflammatory markers based on a single cell type are often susceptible to external fluctuations (38); in contrast, NLR integrates innate and adaptive immune dynamics, enhancing its reliability and interpretability in clinical settings.

We further compared the performance of NLR with CRP and UA in predicting inpatient gout recurrence, revealing that the AUC of NLR surpassed that of CRP and UA. This advantage may stem from NLR’s capacity to reflect both innate and adaptive immune responses (39), thereby capturing more nuanced and sustained inflammatory dynamics than CRP. Moreover, NLR may be more closely aligned to the pathophysiological mechanism of gout, especially considering the key role of neutrophils in the process of gout attack. The CRP is an acute-phase inflammatory biomarker synthesized by the liver in response to immune cytokine stimulation (40). While it exhibits rapid elevation during inflammatory infections and other conditions, its ability to accurately reflect the extent of inflammation often demonstrates a certain time delay, rendering it susceptible to various factors (3). Consistent with our investigation, previous studies have demonstrated that GlycA can serve as a reliable long-term biomarker for assessing the hyperactive state of neutrophils and exhibits superior predictive capability for recurrence compared to UA levels (24). Although several studies have demonstrated a close association between UA levels and the risk of recurrence, it remains challenging to capture the comprehensive inflammatory response of the body solely through direct indicators of uric acid metabolism. Although hyperuricemia is a prerequisite for gout attacks (41), not all patients with hyperuricemia will develop gout, and some gout patients may exhibit normal UA levels during an attack (42, 43). Consequently, the predictive value of UA levels in determining inpatient gout recurrence has certain limitations. Taken together, these results suggest that NLR, as a cost-effective and readily accessible inflammatory marker, outperforms CRP and UA, offering a more comprehensive and pathophysiologically relevant tool for risk stratification and inpatient gout recurrence surveillance in clinical practice. Although the overall discriminative ability of NLR was modest, it still provides meaningful clinical value when interpreted in combination with traditional markers such as UA. The integration of NLR significantly improved model accuracy and net clinical benefit, as confirmed by decision curve analysis, supporting its role as a complementary rather than stand-alone predictor in recurrence risk assessment.

Our study offers novel insights by exploring multiple dimensions of inpatient gout recurrence risk and validating the broad applicability of NLR as a predictive biomarker. Although UA remains the primary biochemical determinant of gout flares, many patients with normal UA levels still experience inpatient gout recurrences, indicating that UA alone may be insufficient for accurate risk stratification. Similarly, tophus formation reflects chronic disease progression, but not all gout patients develop tophus. While ULT is effective in reducing UA levels, its efficacy may be compromised by poor adherence or delayed initiation, and flares can occur even during treatment. Under these conditions, inpatient gout recurrence prediction becomes particularly challenging. Notably, our subgroup analyses demonstrated that NLR consistently predicted inpatient gout recurrence risk across diverse clinical contexts, including patients with normal UA levels, absence of tophus, or ongoing ULT. This highlights the potential of NLR to compensate for the limitations of traditional predictors. We propose that persistent low-grade systemic inflammation may underlie recurrent episodes in such patients, which NLR, by capturing shifts in both neutrophils and lymphocytes, can sensitively detect. Moreover, NLR may help identify subpopulations with subclinical inflammation or inadequate response to ULT. As a composite marker of immune-inflammatory balance, NLR reflects disease heterogeneity and systemic inflammatory burden more comprehensively than single-variable indicators. Its prognostic utility remains stable across subgroups and is minimally influenced by conventional clinical factors, supporting its value as a complementary tool for individualized inpatient gout recurrence risk assessment in patients with gout.

Furthermore, we evaluated the incremental predictive value of incorporating NLR into various machine learning models. The results revealed a notable enhancement in model performance, with the inclusion of NLR significantly improving the accuracy of inpatient gout recurrence prediction. These findings underscore the independent and critical role of NLR in risk stratification and model optimization.

From a clinical perspective, NLR provides a practical, cost-effective, and easily obtainable biomarker that can be incorporated into routine inpatient evaluation for patients with gout. In our study, elevated NLR levels were consistently associated with an increased risk of inpatient gout recurrence, even among patients with normal uric acid levels, absence of tophus, or ongoing urate-lowering therapy. This indicates that NLR can serve as a convenient adjunct for early risk stratification when conventional biochemical markers such as uric acid or CRP fail to fully capture inflammatory activity or inpatient gout recurrence tendency. In practice, measuring NLR at hospital admission could assist clinicians in identifying patients who may benefit from intensified anti-inflammatory prophylaxis, closer monitoring, or adjustment of urate-lowering regimens.

The biological rationale for these findings is supported by the observed pattern of elevated neutrophil and reduced lymphocyte counts in patients with higher NLR, which reflects a systemic pro-inflammatory state and impaired immune regulation. This cellular imbalance aligns with the established pathophysiology of gout, characterized by neutrophil recruitment and IL-1β-mediated inflammasome activation triggered by monosodium urate crystals. Therefore, while NLR should not be interpreted as a stand-alone determinant, its ease of measurement, biological relevance, and consistent association with recurrence risk support its use as a complementary biomarker for individualized inpatient management of gout and for optimizing preventive and therapeutic strategies.

The primary contribution of this study lies in the validation of NLR as a cost-effective, readily accessible, and clinically practical inflammatory biomarker for predicting inpatient gout recurrence. By establishing its utility across diverse cohorts and modeling strategies, our findings provide a more refined basis for clinical decision-making. The integration of NLR into routine assessment could facilitate the development of individualized prevention and treatment strategies, including adjustments to pharmacotherapy and targeted lifestyle interventions, ultimately aiming to reduce the risk of inpatient gout recurrence and improve long-term outcomes in patients with gout.

## Limitations

This study has several limitations. First, our analysis primarily focused on the comparison of NLR, CRP, and UA, without a comprehensive evaluation of other inflammatory markers. Future studies should include a broader range of inflammatory markers to provide a more in-depth comparative analysis. Second, as NLR is a dynamic biomarker influenced by various physiological and pathological conditions, a single time-point measurement may not fully capture the temporal fluctuations relevant to inpatient gout recurrence risk. Future studies should consider longitudinal measurements and larger sample sizes to improve generalizability and model robustness. Third, the exclusion of patients with new gout attacks or those with musculoskeletal diseases requiring NSAIDs or glucocorticoids may limit the generalizability of our findings. While these exclusions were necessary to focus on recurrent gout episodes, they may introduce selection bias, which could affect the applicability of our results to the broader gout population, including those with comorbid conditions or those receiving specific treatments for gout. Additionally, our cohort consisted solely of hospitalized patients, which may limit the external applicability of our findings to outpatient or community-based populations. Further validation is needed to confirm the generalizability of our findings.

## Conclusions

In conclusion, our findings provide robust evidence that NLR is a practical, sensitive, and independently informative biomarker for predicting inpatient gout recurrence. Incorporating NLR into clinical workflows may enable more accurate identification of high-risk individuals, enhance risk stratification and personalized care, and guide optimized treatment decisions to mitigate recurrence risk.

## Data Availability

The raw data supporting the conclusions of this article will be made available by the authors on reasonable request, without undue reservation. Clinical data used in this study were obtained from participating hospitals and, in accordance with institutional regulations and patient-privacy policies, are not publicly available. De-identified clinical data may be shared by the corresponding author upon reasonable request and with appropriate institutional approvals. This study also used the publicly available MIMIC database; access requires completion of the required training and acceptance of the data use agreement. The dataset is available at: https://physionet.org/content/mimiciv.
