# Supplementary material for: Neutrophil-to-lymphocyte ratio predicts inpatient gout recurrence: a large-scale multicenter retrospective cohort with machine-learning validation
Source: Front Immunol. 2025 Nov 10;16:1688516. doi: 10.3389/fimmu.2025.1688516 (PMC12640940; doi:10.3389/fimmu.2025.1688516)
Supplement: Supplementary file 1 [file DataSheet1.pdf]

SUPPLEMENTARY MATERIALS

SUPPLEMENTARY MATERIALS 1
Supplementary Methods 2
GoutRe Data Cleaning Process 2
MIMIC-IV Data Cleaning Process 2
Assessment of Covariates in the MIMIC-IV Cohort 3
Supplementary Tables 4
eTable 1.Classification of the Admission Diagnosis and Source of the Patient Population 4
eTable 2. Candidate Variables 5
eTable 3. Definitions of Variables in GoutRe Cohort (Including ICD-10 Codes for Disease History) 6
eTable 4. Diagnostic ICD Codes in MIMIC-IV Cohort 7
eTable 5. Summary of Variables and Percentage of Missing Data in the GoutRe and MIMIC-IV Cohorts 10
eTable 6. R environment and packages used for machine-learning and statistical analyses 11
eTable 7. Hyperparameters and optimization strategy 11
eTable 8. Multivariate Cox Regression Analysis of Factors Associated with Inpatient Gout Recurrence in GoutRe and MIMIC-IV Cohorts 12
eTable 9. Multivariate-Adjusted Hazard Ratios (95% CI) of NLR for Gout Inpatient Gout Recurrence With Normal Serum Uric Acid 13
eTable 10. Multivariate-Adjusted Hazard Ratios (95% CI) of NLR for Inpatient Gout Recurrence Without Tophus 14
eTable 11. Multivariate-Adjusted Hazard Ratios (95% CI) of NLR for Inpatient Gout Recurrence Without Tophus and Normal Serum Uric Acid 15
eTable 12. Multivariate-Adjusted Hazard Ratios (95% CI) of NLR for Inpatient Gout Recurrence With Urate-lowering Therapy 16
eTable 13. Baseline Characteristics 17
eTable 14. Improved Discrimination and Risk Reclassification for Inpatient Gout Recurrence With or Without NLR 19
Supplementary Figures 20
eFigure 1. Overview of Study Design 20
eFigure 2. Correlation and ROC Curve Analyses Between NLR and CRP for Predicting Inpatient Gout Recurrence 21
eFigure 3. Kaplan-Meier Survival Curve for Inpatient Gout Recurrence in Subgroup in the GoutRe Cohort and the MIMIC-IV Cohort 22
eFigure 4. Kaplan-Meier Survival Curve for Inpatient Gout Recurrence in Subgroup Under Contrasting Conditions in the GoutRe Cohort and the MIMIC-IV Cohort 23
eFigure 5. Cumulative Incidence of Inpatient Gout Recurrence in Subgroup in the GoutRe Cohort and the MIMIC-IV Cohort 24
eFigure 6. Cumulative Incidence of Inpatient Gout Recurrence in Subgroup Under Contrasting Conditions in the GoutRe Cohort and the MIMIC-IV Cohort25
eFigure 7. Decision Curve Analysis for Inpatient Gout Recurrence Prediction Models in the GoutRe and MIMIC-IV Cohorts 26
REFERENCES： 27

Supplementary Methods

GoutRe Data Cleaning Process

Data from the Gout Recurrence (GoutRe) multicentre cohort were extracted from the electronic medical record systems of five tertiary hospitals in China using a unified data dictionary and standardized protocols. Laboratory variables were normalized to consistent units and reference ranges; unit discrepancies (e.g., plateletcrit reported as “%” or “mL/L”) were resolved through conversion tables. Outliers exceeding physiologic thresholds were truncated, and qualitative results were converted into standardized quantitative scales. For parameters with multiple reporting standards (e.g., RDW-SD vs RDW-CV), values were harmonized to a unified format.

After harmonization, all records underwent cross-hospital consistency checks for variable definitions, missingness, and implausible values to ensure data reliability.

MIMIC-IV Data Cleaning Process

Access to the MIMIC-IV database was obtained through a comprehensive learning and testing process. We collected related data from the MIMIC-IV database using Structured Query Language performed on Navicat Premium ver. 16.3.2[1]. Initially, patients diagnosed with gout in the "diagnoses_icd" table were identified using ICD codes (eTable 3). We first collected the ICD codes for gout from the "d_ICD_diagnoses" table, and then retrieved the patient data for all patients with corresponding diagnoses from the "diagnoses_icd" table, totaling 18,946 patients. All patient data including demographic information, lifestyle factors, laboratory results, comorbidities, and medication details were collected from tables such as "admissions", "omr", "labevents", "patients", "prescriptions", and "diagnoses_icd" using their respective hadm-id and/(or) subject-id[2]. Race, admission location, admission time, and discharge time were collected from the "admissions" table. Anchor age and Sex were collected from the "patients" table. The laboratory tests for WBC, NEUT, LYMPH, ESR, UA, eGFR, and CRP were first identified by their corresponding item IDs in the "d_labitems" table and then searched and sorted in the "labevents" table.

The ICD codes for all comorbidities were collected from the "d_icd_diagnosis" table, and then the corresponding diagnostic data for comorbidities in gout patients

were collected from the "diagnoses_icd" table. All comorbidities were grouped into their respective categories[3, 4](eTable 2), regardless of whether the patient had one or more of these conditions. For example, chronic kidney disease was categorized as Renal disease[5]. Similarly, medication information was also summarized. We collected all medication information for gout patients using the hadm-id. To obtain a more comprehensive understanding of their medication use, we attempted to collect the GSN code for each medication and searched and summarized it in the "prescriptions" table. Each drug was then categorized according to its corresponding category[6].

Assessment of Covariates in the MIMIC-IV Cohort

In the GoutRe cohort, all patients were of Chinese ethnicity. In the MIMIC-IV cohort, race was categorized into Asian (Asian Indian, Chinese, Korean, Southeast Asian, and other), American Indian or Alaska Native, White (Brazilian, Eastern European, Russian, and other European), Hispanic or Latino (Central American, Colombian, Cuban, Dominican, Guatemalan, Honduran, Mexican, Puerto Rican, Salvadoran), Portuguese, Black (African, African American, Cape Verdean, Caribbean Islander), Native Hawaiian or other Pacific Islander, multiple race and ethnicity, and other. Medications were that have been reported to be associated with gout attacks or to affect fluctuations in blood Uric acid levels[7, 8]. Benzbromarone was used in domestic clinical practice, while it has been withdrawn from the market in the United States. Cardiovascular diseases included angina pectoris and coronary atherosclerotic heart disease. Kidney disease included renal insufficiency, kidney failure,chronic kidney disease,and uremia. Thyroid diseases included hyperthyroidism, hypothyroidism,thyroid nodules, thyroid cysts, and thyroid tumors. Dyslipidemia refers to elevated or decreased levels of total cholesterol (TC), triglyceride (TG), low-density lipoprotein cholesterol (LDL-C), high-density lipoprotein cholesterol (HDL-C), and/(or) lipoprotein-α (LPα) in the plasma. Stroke included cerebral hemorrhage and cerebral infarction.

Supplementary Tables

eTable 1.Classification of the Admission Diagnosis and Source of the Patient Population

	MIMIC-IV cohort		GoutRe cohort	
	**Count**	**Percentage**	**Count**	**Percentage**
**Classification of diagnosis**				
Disease of endocrine and metabolic systems	81	4.01%	353	6.32%
Diseases of circulatory system	554	27.44%	1065	19.07%
Diseases of digestive system	243	12.04%	583	10.44%
Diseases of musculoskeletal system	132	6.54%	213	3.81%
Diseases of nervous system	123	6.09%	618	11.07%
Diseases of reproductive system	4	0.20%	23	0.41%
Diseases of respiratory system	137	6.79%	235	4.21%
Diseases of sense organ	1	0.05%	115	2.06%
Diseases of the blood	181	8.96%	108	1.93%
Diseases of the skin	13	0.64%	57	1.02%
Diseases of urinary system	119	5.89%	1108	19.84%
Infectious disease	182	9.01%	197	3.53%
Injury/External causes	65	3.22%	245	4.39%
Neoplasm	95	4.71%	413	7.40%
Others	89	4.41%	251	4.49%
**Source of the patient population**				
Ambulatory surgery transfer	1	0.05%	NA	NA
Clinic referral	75	3.71%	NA	NA
Emergency room	960	47.55%	NA	NA
Information not available	3	0.15%	NA	NA
Internal transfer to or from the psychology	7	0.35%	NA	NA
Post anesthesia care unit（PACU）	8	0.40%	NA	NA
Physician referral	519	25.71%	NA	NA
Procedures site	22	1.09%	NA	NA
Transfer from hospital	309	15.30%	NA	NA
Transfer from skilled nursing facility	39	1.93%	NA	NA
Walk in or self referral	76	3.76%	NA	NA

eTable 2. Candidate Variables

Domains	Variables	
Demographic	Age, Race, Sex, Weight changed	
Lifestyle factors	Smoking history, Drinking history	
Laboratory	Complete blood count	WBC, NEUT, LYMPH
	Kidney function	UA, eGFR
Physical examination	Tophus	
Comorbidities	Diabetes, Hypertension, Cardiovascular disease, Heart failure, Stroke, Dyslipidemia, Fatty liver, Renal disease, Thyroid disease, Cancer, Stones, MetS	
Medication	Anti-gout drug	Urate-lowering therapy(Allopurinol, Febuxostat, Benzbromarone)[a], $NaHCO_3$, GCs
	Hypoglycemic agent	Sodium-glucose co-transporter 2 (SGLT2) inhibitors
	Cardiovascular drugs	CCB, Losartan, NL-ARBs, β-blockers, Diuretic
	Anticoagulants	Low-dose Aspirin (ASA)[b]
	Lipid-modifying agents	Statins, Fenofibrate
	Mannitol	

Abbreviations: WBC, White blood cells; NEUT, Neutrophils; LYMPH, Lymphocytes; UA,Uric acid; eGFR, Estimated glomerular filtration rate; MetS, Metabolic syndrome; $NaHCO_3$, Sodium bicarbonate; GCs, Glucocorticoids; SGLT2 inhibitors, Sodium-glucose co-transporter 2 inhibitors; CCB, Calcium channel blockers; NL-ARBs, Non-losartan angiotensin II receptor blockers; β-blocker, Beta-blocker; ASA, Acetylsalicylic acid.

[a] The GoutRe cohort included Allopurinol, Benzbromarone, and Febuxostat as Urate-lowering therapy; while the MIMIC cohort excluded Benzbromarone.

[b] Low dose refers to less than 100mg.

eTable 3. Definitions of Variables in GoutRe Cohort (Including ICD-10 Codes for Disease History)

Variables	Definition
Weight changed	Any weight gain or loss in previous medical history compared with the last hospitalization or in the past six months.
Metabolic syndrome, MetS	Metabolic disorders diagnosed simultaneously with Hypertension, Diabetes and Dyslipidemia.
Diabetes mellitus	ICD-10: E10-E14, including Type 1 diabetes mellitus, Type 2 diabetes mellitus, Malnutrition-related diabetes mellitus, Other specified diabetes mellitus and Unspecified diabetes mellitus.
Hypertension	ICD-10: I10-I15, According to the Chinese Clinical Practice Guidelines for Hypertension, the diagnostic criteria for adult Hypertension are systolic blood pressure ≥130mmHg and/or diastolic blood pressure ≥80mmHg.
Cardiovascular diseases	Heart diseases and Coronary arteriovenous diseases caused by various causes, such as Angina pectoris (ICD-10:I20), Coronary atherosclerotic heart disease (ICD-10:I25.1), Heart failure (ICD-10:I50) , etc.
Heart failure	ICD-10:I50, mainly including Congestive heart failure, Left ventricular failure.
Stroke	Local brain dysfunction caused by acute cerebrovascular disease including Cerebral hemorrhage (ICD-10:I61) or Cerebral infarction (ICD-10:I63).
Dyslipidemia	Abnormal blood lipids mainly referring to elevated or decreased levels of Total cholesterol (TC),Triglyceride (TG), Low-density lipoprotein cholesterol (LDL-C), High-density lipoprotein cholesterol (HDL-C), and/(or) Lipoprotein-α(LPα) in the plasma (ICD-10:E78).
Fatty liver	A liver disease including Alcoholic fatty liver(ICD-10: K70.0) and Fatty (change of) liver, not elsewhere classified (ICD-10:K76.0).
Renal diseases	Diagnosed with Renal insufficiency (ICD-10:N18.0,N19), Kidney failure (ICD-10:N17-N18), Chronic kidney disease (ICD-10:N18.9), and Uremia (ICD-10:N19.x01).
Thyroid diseases	A range of diseases caused by thyroid dysfunction including Hyperthyroidism (ICD-10:E05.901), Hypothyroidism (ICD-10:E00-E03), Thyroid nodules (ICD-10:E04-E05), Thyroid cysts (ICD:E07.0), and Thyroid tumors (ICD-10:C73), etc.
Cancer	ICD-10: C00-C14, Malignant neoplasms of lip, oral cavity and pharynx<br>ICD-10:C00-C75, Malignant neoplasms, stated or presumed to be primary, of specified sites, except of lymphoid, haematopoietic and related tissue.
Stones	A disease refers to solid deposits of inorganic or organic substances that form in the cavities of the kidneys, ureters, gallbladder, or bladder, including Urolithiasis (ICD-10:N20-N23), Calculus of prostate(ICD-10:N42.0), Calculus of kidney (ICD-10:N20.0), Gallstone pancreatitis(ICD-10:N20.0), etc.
Tophus	Deposit crystals of sodium urate (typically located in joints, ears, olecranon bursae, finger pads, and tendons), often accompanied by vascular coverage reported positively on physical examination.
Gout	ICD-10:M10, a disease caused by excessive deposits of uric acid.

eTable 4. Diagnostic ICD Codes in MIMIC-IV Cohort

Features	Classification under features	ICD-code
Metabolic syndrome		2777,E8881
Diabetes mellitus		24900,24901,24910,24911,24920,24921,24930,24931,24940,24941,24950,24951,24960,24961,24970,24971,24980,24981,24990,24991,25000,25001,25002,25003,25010,25011,25012,25013,25020,25021,25022,25023,25030,25031,25032,25033,25040,25041,25042,25043,25050,25051,25052,25053,25060,25061,25062,25063,25070,25071,25072,25073,25080,25081,25082,25083,25090,25091,25092,25093,E08,E080,E0800,E0801,E081,E0810,E0811,E082,E0821,E0822,E0829,E083,E0831,E08311,E08319,E0832,E08321,E083211,E083212,E083213,E083219,E08329,E083291,E083292,E083293,E083299,E0833,E08331,E083311,E083312,E083313,E083319,E08339,E083391,E083392,E083393,E083399,E0834,E08341,E083411,E083412,E083413,E083419,E08349,E083491,E083492,E083493,E083499,E0835,E08351,E083511,E083512,E083513,E083519,E08352,E083521,E083522,E083523,E083529,E08353,E083531,E083532,E083533,E083539,E08354,E083541,E083542,E083543,E083549,E08355,E083551,E083552,E083553,E083559,E08359,E083591,E083592,E083593,E083599,E0836,E0837,E0837X1,E0837X2,E0837X3,E0837X9,E0839,E084,E0840,E0841,E0842,E0843,E0844,E0849,E085,E0851,E0852,E0859,E086,E0861,E08610,E08618,E0862,E08620,E08621,E08622,E08628,E0863,E08630,E08638,E0864,E08641,E08649,E0865,E0869,E088,E089,E09,E090,E0900,E0901,E091,E0910,E0911,E092,E0921,E0922,E0929,E093,E0931,E09311,E09319,E0932,E09321,E093211,E093212,E093213,E093219,E09329,E093291,E093292,E093293,E093299,E0933,E09331,E093311,E093312,E093313,E093319,E09339,E093391,E093392,E093393,E093399,E0934,E09341,E093411,E093412,E093413,E093419,E09349,E093491,E093492,E093493,E093499,E0935,E09351,E093511,E093512,E093513,E093519,E09352,E093521,E093522,E093523,E093529,E09353,E093531,E093532,E093533,E093539,E09354,E093541,E093542,E093543,E093549,E09355,E093551,E093552,E093553,E093559,E09359,E093591,E093592,E093593,E093599,E0936,E0937,E0937X1,E0937X2,E0937X3,E0937X9,E0939,E094,E0940,E0941,E0942,E0943,E0944,E0949,E095,E0951,E0952,E0959,E096,E0961,E09610,E09618,E0962,E09620,E09621,E09622,E09628,E0963,E09630,E09638,E0964,E09641,E09649,E0965,E0969,E098,E099,E10,E101,E1010,E1011,E102,E1021,E1022,E1029,E103,E1031,E10311,E10319,E1032,E10321,E103211,E103212,E103213,E103219,E10329,E103291,E103292,E103293,E103299,E1033,E10331,E103311,E103312,E103313,E103319,E10339,E103391,E103392,E103393,E103399,E1034,E10341,E103411,E103412,E103413,E103419,E10349,E103491,E103492,E103493,E103499,E1035,E10351,E103511,E103512,E103513,E103519,E10352,E103521,E103522,E103523,E103529,E10353,E103531,E103532,E103533,E103539,E10354,E103541,E103542,E103543,E103549,E10355,E103551,E103552,E103553,E103559,E10359,E103591,E103592,E103593,E103599,E1036,E1037,E1037X1,E1037X2,E1037X3,E1037X9,E1039,E104,E1040,E1041,E1042,E1043,E1044,E1049,E105,E1051,E1052,E1059,E106,E1061,E10610,E10618,E1062,E10620,E10621,E10622,E10628,E1063,E10630,E10638,E1064,E10641,E10649,E1065,E1069,E108,E109,E11,E110,E1100,E1101,E111,E1110,E1111,E112,E1121,E1122,E1129,E113,E1131,E11311,E11319,E1132,E11321,E113211,E113212,E113213,E113219,E11329,E113291,E113292,E113293,E113299,E1133,E11331,E113311,E113312,E113313,E113319,E11339,E113391,E113392,E113393,E113399,E1134,E11341,E113411,E113412,E113413,E113419,E11349,E113491,E113492,E113493,E113499,E1135,E11351,E113511,E113512,E113513,E113519,E11352,E113521,E113522,E113523,E113529,E11353,E113531,E113532,E113533,E113539,E11354,E113541,E113542,E113543,E113549,E11355,E113551,E113552,E113553,E113559,E11359,E113591,E113592,E113593,E113599,E1136,E1137,E1137X1,E1137X2,E1137X3,E1137X9,E1139,E114,E1140,E1141,E1142,E1143,E1144,E1149,E115,E1151,E1152,E1159,E116,E1161,E11610,E11618,E1162,E11620,E11621,E11622,E11628,E1163,E11630,E11638,E1164,E11641,E11649,E1165,E1169,E118,E119,E13,E130,E1300,E1301,E131,E1310,E1311,E132,E1321,E1322,E1329,E133,E1331,E13311,E13319,E1332,E13321,E133211,E133212,E133213,E133219,E13329,E133291,E133292,E133293,E133299,E1333,E13331,E133311,E133312,E133313,E133319,E13339,E133391,E133392,E133393,E133399,E1334,E13341,E133411,E133412,E133413,E133419,E13349,E133491,E133492,E133493,E133499,E1335,E13351,E133511,E133512,E133513,E133519,E13352,E133521,E133522,E133523,E133529,E13353,E133531,E133532,E133533,E133539,E13354,E133541,E133542,E133543,E133549,E13355,E133551,E133552,E133553,E133559,E13359,E133591,E133592,E133593,E133599,E1336,E1337,E1337X1,E1337X2,E1337X3,E1337X9,E1339,E134,E1340,E1341,E1342,E1343,E1344,E1349,E135,E1351,E1352,E1359,E136,E1361,E13610,E13618,E1362,E13620,E13621,E13622,E13628,E1363,E13630,E13638,E1364,E13641,E13649,E1365,E1369,E138,E139,E710,O24,O240,O2401,O24011,O24012,O24013,O24019,O2402,O2403,O241,O2411,O24111,O24112,O24113,O24119,O2412,O2413,O244,O2441,O24410,O24414,O24415,O24419,O2442,O24420,O24424,O24425,O24429,O2443,O24430,O24434,O24435,O24439,O248,O2481,O24811,O24812,O24813,O24819,O2482,O2483,O249,P702,R7303,V1221,Z8631,Z8632
Hypertension		4010,4011,4019,40501,40509,40511,40519,40591,40599,4160,45930,45931,45932,45933,45939,5723,64200,64201,64202,64203,64204,64210,64211,64212,64213,64214,64220,64221,64222,64223,64224,64230,64231,64232,64233,64234,64270,64271,64272,64273,64274,64290,64291,64292,64293,64294,99791,I10,I15,I150,I151,I152,I158,I159,I270,I272,I2720,I2721,I2722,I2723,I2724,I2729,I873,I8730,I87301,I87302,I87303,I87309,I8731,I87311,I87312,I87313,I87319,I8732,I87321,I87322,I87323,I87329,I8733,I87331,I87332,I87333,I87339,I8739,I87391,I87392,I87393,I87399,I973,O10,O100,O1001,O10011,O10012,O10013,O10019,O1002,O1003,O104,O1041,O10411,O10412,O10413,O10419,O1042,O1043,O109,O1091,O10911,O10912,O10913,O10919,O1092,O11,O111,O112,O113,O114,O115,O119,O13,O131,O132,O133,O134,O135,O139,O16,O161,O162,O163,O164,O165,O169,P292,P2930
Cardiovascular disease	Angina pectoris	4139,I20,I201,I208,I209,I2510,I2511,I25110,I25111,I25118,I25119,I257,I2570,I25700,I25701,I25708,I25709,I2571,I25710,I25711,I25718,I25719,I2572,I25720,I25721,I25728,I25729,I2573,I25730,I25731,I25738,I25739,I2575,I25751,I25758,I25759,I2576,I25761,I25768,I25769,I2579,I25790,I25791,I25798,I25799
	Coronary atherosclerotic heart disease	41400,41401,41402,41403,41404,41405,41406,41407,4143,4144,I2511,I25110,I2575,I25750,I25751,I25758,I25759,I25811,I2583,I2584
Heart failure		4281,I5081,I50810,I50811,I50812,I50813,I50814
Stroke	Cerebral hemorrhage	431,7670,I61,I610,I611,I612,I613,I614,I615,I616,I618,I619,P101,S0636,S06360,S06360A,S06360D,S06360S,S06361,S06361A,S06361D,S06361S,S06362,S06362A,S06362D,S06362S,S06363,S06363A,S06363D,S06363S,S06364,S06364A,S06364D,S06364S,S06365,S06365A,S06365D,S06365S,S06366,S06366A,S06366D,S06366S,S06367,S06367A,S06367D,S06367S,S06368,S06368A,S06368D,S06368S,S06369,S06369A,S06369D,S06369S
	Cerebral infarction	34660,34661,34662,34663,43311,43321,43381,43401,43411,43491,G436,G4360,G43601,G4361,G43611,G43619,I63,I6300,I6301,I63011,I63019,I6303,I63031,I6311,I63119,I6312,I6313,I63131,I63132,I63133,I6319,I6321,I63211,I6323,I63233,I63239,I6329,I633,I6331,I63313,I63323,I63329,I6333,I63331,I63332,I63333,I63349,I634,I6340,I63421,I63422,I63423,I63429,I63439,I6344,I63443,I63449,I6349,I635,I63519,I63522,I63523,I63533,I63539,I6354,I63543,I638,I6381,I6389,I639,V1254,Z8673
Dyslipidemia	Hyperlipidemia	2722,2724,E782,E784,E7849,E785
	Hypercholesterolemia	2720,38582,5756,E780,E7800,E7801,E787,E7870,E7879,K824
	Lipoprotein	2725,E78,E7841,E786,E788,E7889,E789
Fatty liver		5710,K700,K760
Renal disease	Kidney failure	5845,5846,5848,5849,6393,66930,66932,66934,N17,N170,N171,N172,N178,N179,N19,N990,O0332,O0382,O0482,O0732,O084,O904
	Chronic kidney disease	40300,40301,40310,40311,40390,40391,40400,40401,40402,40403,40410,40411,40412,40413,40490,40491,40492,40493,5851,5852,5853,5854,5855,5859,D631,E0922,E1022,E1122,E1322,I12,I120,I129,I13,I130,I131,I1310,I1311,I132,N18,N181,N182,N183,N184,N185,N189
Renal disease	Uremia	28311,D593,R392
Thyroid disease	Hyperthyroidism	E05,P721
	Hypothyroidism	243,2440,2441,2442,2443,2448,2449,E02,E03,E030,E031,E032,E033,E038,E039,E890
	Thyroid nodule	24240,24241,E041,E051,E0510,E0511
	Thyroid cysts	2462

	Thyroid tumors	C73
Cancer	Malignant tumor	1400,1401,1403,1404,1405,1406,1408,1409,1410,1411,1412,1413,1414,1415,1416,1418,1419,1420,1421,1422,1428,1429,1430,1431,1438,1439,1440,1441,1448,1449,1450,1451,1452,1453,1454,1455,1456,1458,1459,1460,1461,1462,1463,1464,1465,1466,1467,1468,1469,1470,1471,1472,1473,1478,1479,1480,1481,1482,1483,1488,1489,1490,1491,1498,1499,1500,1501,1502,1503,1504,1505,1508,1509,1510,1511,1512,1513,1514,1515,1516,1518,1519,1520,1521,1522,1523,1528,1529,1530,1531,1532,1533,1534,1535,1536,1537,1538,1539,1540,1541,1542,1543,1548,1550,1551,1552,1560,1561,1562,1568,1569,1570,1571,1572,1573,1574,1578,1579,1580,1588,1589,1590,1591,1598,1599,1600,1601,1602,1603,1604,1605,1608,1609,1610,1611,1612,1613,1618,1619,1620,1622,1623,1624,1625,1628,1629,1630,1631,1638,1639,1640,1641,1642,1643,1648,1649,1650,1658,1659,1700,1701,1702,1703,1704,1705,1706,1707,1708,1709,1710,1712,1713,1714,1715,1716,1717,1718,1719,1730,17300,17309,1731,17310,17319,1732,17320,17329,1733,17330,17339,1734,17340,17349,1735,17350,17359,1736,17360,17369,1737,17370,17379,1738,17380,17389,1739,17390,17399,1740,1741,1742,1743,1744,1745,1746,1748,1749,1750,1759,179,1800,1801,1808,1809,181,1820,1821,1828,1830,1832,1833,1834,1835,1838,1839,1840,1841,1842,1843,1844,1848,1849,185,1860,1869,1871,1872,1873,1874,1875,1876,1877,1878,1879,1880,1881,1882,1883,1884,1885,1886,1887,1888,1889,1890,1891,1892,1893,1894,1898,1899,1900,1901,1902,1903,1904,1905,1906,1907,1908,1899,1900,1901,1902,1903,1904,1905,1906,1907,1908,1919,1920,1921,1922,1923,1928,1929,193,1940,1941,1943,1944,1945,1946,1948,1949,1950,1951,1952,1953,1954,1955,1958,1960,1961,1962,1963,1965,1966,1968,1969,1970,1971,1972,1973,1974,1975,1976,1977,1978,1980,1981,1982,1983,1984,1985,1986,1987,19881,19882,19889,1990,1991,1992,20290,20291,20292,20293,20294,20295,20296,20297,20298,79506,79516,79676,C00,C000,C001,C002,C003,C004,C005,C006,C008,C009,C01,C02,C020,C021,C022,C023,C024,C028,C029,C03,C030,C031,C039,C04,C040,C041,C048,C049,C05,C050,C051,C052,C058,C059,C06,C060,C061,C062,C068,C0680,C0689,C069,C07,C08,C080,C081,C089,C09,C090,C091,C098,C099,C10,C100,C101,C102,C103,C104,C108,C109,C11,C110,C111,C112,C113,C118,C119,C12,C13,C130,C131,C132,C138,C139,C14,C140,C142,C148,C15,C153,C154,C155,C158,C159,C16,C160,C161,C162,C163,C164,C165,C166,C168,C169,C17,C170,C171,C172,C178,C179,C18,C180,C181,C182,C183,C184,C185,C186,C187,C188,C189,C19,C20,C21,C210,C211,C212,C218,C22,C228,C229,C23,C24,C240,C241,C248,C249,C25,C250,C251,C252,C253,C254,C257,C258,C259,C26,C260,C261,C269,C30,C300,C301,C31,C310,C311,C312,C313,C318,C319,C32,C320,C321,C322,C323,C328,C329,C33,C34,C340,C3400,C3401,C3402,C341,C3410,C3411,C3412,C342,C343,C3430,C3431,C3432,C348,C3480,C3481,C3482,C349,C3490,C3491,C3492,C37,C38,C380,C381,C382,C383,C384,C388,C39,C390,C399,C40,C400,C4000,C4001,C4002,C401,C4010,C4011,C4012,C402,C4020,C4021,C4022,C403,C4030,C4031,C4032,C408,C4080,C4081,C4082,C409,C4090,C4091,C4092,C41,C410,C411,C412,C413,C414,C419,C44,C440,C4400,C4409,C441,C4410,C44101,C44102,C441021,C441022,C44109,C441091,C441092,C4419,C44191,C44192,C441921,C441922,C44199,C441991,C441992,C442,C4420,C44201,C44202,C44209,C4429,C44291,C44292,C44299,C443,C4430,C44300,C44301,C44309,C4439,C44390,C44391,C44399,C444,C4440,C4449,C445,C4450,C44500,C44501,C44509,C4459,C44590,C44591,C44599,C446,C4460,C44601,C44602,C44609,C4469,C44691,C44692,C44699,C447,C4470,C44701,C44702,C44709,C4479,C44791,C44792,C44799,C448,C4480,C4489,C449,C4490,C4499,C47,C470,C471,C4710,C4711,C4712,C472,C4720,C4721,C4722,C473,C474,C475,C476,C478,C479,C48,C480,C481,C482,C488,C49,C490,C491,C4910,C4911,C4912,C492,C4920,C4921,C4922,C493,C494,C495,C496,C498,C499,C50,C500,C5001,C50011,C50012,C50019,C5002,C50021,C50022,C50029,C501,C5011,C50111,C50112,C50119,C5012,C50121,C50122,C50129,C502,C5021,C50211,C50212,C50219,C5022,C50221,C50222,C50229,C503,C5031,C50311,C50312,C50319,C5032,C50321,C50322,C50329,C504,C5041,C50411,C50412,C50419,C5042,C50421,C50422,C50429,C505,C5051,C50511,C50512,C50519,C5052,C50521,C50522,C50529,C506,C5061,C50611,C50612,C50619,C5062,C50621,C50622,C50629,C508,C5081,C50811,C50812,C50819,C5082,C50821,C50822,C50829,C509,C5091,C50911,C50912,C50919,C5092,C50921,C50922,C50929,C51,C510,C511,C512,C518,C519,C52,C53,C530,C531,C538,C539,C54,C540,C541,C542,C543,C548,C549,C55,C56,C561,C562,C569,C57,C570,C5700,C5701,C5702,C571,C5710,C5711,C5712,C572,C5720,C5721,C5722,C573,C574,C577,C578,C579,C58,C60,C600,C601,C602,C608,C609,C61,C62,C620,C6200,C6201,C6202,C621,C6210,C6211,C6212,C629,C6290,C6291,C6292,C63,C630,C6300,C6301,C6302,C631,C6310,C6311,C6312,C632,C637,C638,C639,C64,C641,C642,C649,C65,C651,C652,C659,C66,C661,C662,C669,C67,C670,C671,C672,C673,C674,C675,C676,C677,C678,C679,C68,C680,C681,C688,C689,C69,C690,C6900,C6901,C6902,C691,C6910,C6911,C6912,C692,C6920,C6921,C6922,C693,C6930,C6931,C6932,C694,C6940,C6941,C6942,C695,C6950,C6951,C6952,C696,C6960,C6961,C6962,C698,C6980,C6981,C6982,C699,C6990,C6991,C6992,C70,C700,C701,C709,C71,C710,C711,C712,C713,C714,C715,C716,C717,C718,C719,C72,C720,C721,C722,C7220,C7221,C7222,C723,C7230,C7231,C7232,C724,C7240,C7241,C7242,C725,C7250,C7259,C729,C73,C74,C740,C7400,C7401,C7402,C741,C7410,C7411,C7412,C749,C7490,C7491,C7492,C75,C750,C751,C752,C753,C754,C755,C758,C759,C76,C760,C761,C762,C763,C764,C7640,C7641,C7642,C765,C7650,C7651,C7652,C768,C77,C770,C771,C772,C773,C774,C775,C778,C779,C78,C780,C7800,C7801,C7802
	Carcinoma	17301,17302,17311,17312,17321,17322,17331,17332,17341,17342,17351,17352,17361,17362,17371,17372,17381,17382,17391,17392,20900,20901,20902,20903,20910,20911,20912,20913,20914,20915,20916,20917,20920,20921,20922,20923,20924,20925,20926,20927,20929,20930,20931,20932,20933,20934,20935,20936,20940,20941,20942,20943,20950,20951,20975,2300,2301,2302,2303,2304,2305,2306,2307,2308,2309,2310,2311,2312,2318,2319,2320,2321,2322,2323,2324,2325,2326,2327,2328,2329,2330,2331,2332,23330,23331,23332,23339,2334,2335,2336,2337,2339,2340,2348,2349,5281,C220,C221,C227,C4401,C4402,C4411,C44111,C44112,C441121,C441122,C44119,C441191,C441192,C4412,C44121,C44122,C441221,C441222,C44129,C441291,C441292,C4413,C44131,C44132,C441321,C441322,C44139,C441391,C441392,C4421,C44211,C44212,C44219,C4422,C44221,C44222,C44229,C4431,C44310,C44311,C44319,C4432,C44320,C44321,C44329,C4441,C4442,C4451,C44510,C44511,C44519,C4452,C44520,C44521,C44529,C4461,C44611,C44612,C44619,C4462,C44621,C44622,C44629,C4471,C44711,C44712,C44719,C4472,C44721,C44722,C44729,C4481,C4482,C4491,C4492,C4A,C4A0,C4A1,C4A10,C4A11,C4A111,C4A112,C4A12,C4A121,C4A122,C4A2,C4A20,C4A21,C4A22,C4A3,C4A30,C4A31,C4A39,C4A4,C4A5,C4A51,C4A52,C4A59,C4A6,C4A60,C4A61,C4A62,C4A7,C4A70,C4A71,C4A72,C4A8,C4A9,C7A0,C7A00,C7A01,C7A010,C7A011,C7A012,C7A019,C7A02,C7A020,C7A021,C7A022,C7A023,C7A024,C7A025,C7A026,C7A029,C7A09,C7A090,C7A091,C7A092,C7A093,C7A094,C7A095,C7A096,C7A098,C7B0,C7B00,C7B01,C7B02,C7B03,C7B04,C7B09,C7B1,D00,D000,D0000,D0001,D0002,D0003,D0004,D0005,D0006,D0007,D0008,D001,D002,D01,D010,D011,D012,D013,D014,D0140,D0149,D015,D017,D019,D02,D020,D021,D022,D0220,D0221,D0222,D023,D024,D04,D040,D041,D0410,D0411,D04111,D04112,D0412,D04121,D04122,D042,D0420,D0421,D0422,D043,D0430,D0439,D044,D045,D046,D0460,D0461,D0462,D047,D0470,D0471,D0472,D048,D049,D05,D050,D0500,D0501,D0502,D051,D0510,D0511,D0512,D058,D0580,D0581,D0582,D059,D0590,D0591,D0592,D06,D060,D061,D067,D069,D07,D070,D071,D072,D073,D0730,D0739,D074,D075,D076,D0760,D0761,D0769,D09,D090,D091,D0910,D0919,D092,D0920,D0921,D0922,D093,D098,D099,Z85020,Z85030,Z85040,Z85060,Z85110,Z85230,Z85520,Z85821
Stones		27411,37254,5275,57400,57401,57410,57411,57420,57421,57430,57431,57440,57441,57450,57451,57460,57461,57470,57471,57480,57481,57490,57491,5920,5921,5929,5940,5941,5942,5948,5949,6020,H1112,H11121,H11122,H11123,H11129,K115,K800,K8000,K8001,K801,K8010,K8011,K8012,K8013,K8018,K8019,K802,K8020,K8021,K803,K8030,K8031,K8032,K8033,K8034,K8035,K8036,K8037,K804,K8040,K8041,K8042,K8043,K8044,K8045,K8046,K8047,K805,K8050,K8051,K806,K8060,K8061,K8062,K8063,K8064,K8065,K8066,K8067,K807,K8070,K8071,N132,N20,N200,N201,N202,N209,N21,N210,N211,N218,N219,N22,N420,V1301,Z87442
Tophus		27403,27481,27482,M1A00X1,M1A0121,M1A0191,M1A0211,M1A0221,M1A0291,M1A0121,M1A0191,M1A0211,M1A0221,M1A0291,M1A0491,M1A0511,M1A0521,M1A0591,M1A0611,M1A0621,M1A0691,M1A0711,M1A0721,M1A0791,M1A08X1,M1A09X1,M1A10X1,M1A1111,M1A1121,M1A1191,M1A1211,M1A1221,M1A1291,M1A1311,M1A1321,M1A1391,M1A1411,M1A1421,M1A1491,M1A1511,M1A1521,M1A1591,M1A1611,M1A1621,M1A1691,M1A1711,M1A1721,M1A1791,M1A18X1,M1A19X1,M1A20X1,M1A2111,M1A2121,M1A2191,M1A2211,M1A2221,M1A2291,M1A2311,M1A2321,M1A2391,M1A2411,M1A2421,M1A2491,M1A2511,M1A2521,M1A2591,M1A2611,M1A2621,M1A2691,M1A2711,M1A2721,M1A2791,M1A28X1,M1A29X1,M1A30X1,M1A3111,M1A3121,M1A3191,M1A3211,M1A3221,M1A3291,M1A3311,M1A3321,M1A3391,M1A3411,M1A3421,M1A3491,M1A3511,M1A3521,M1A3591,M1A3611,M1A3621,M1A3691,M1A3711,M1A3721,M1A3791,M1A38X1,M1A39X1,M1A40X1,M1A4111,M1A4121,M1A4191,M1A4211,M1A4221,M1A4291,M1A4311,M1A4321,M1A4391,M1A4411,M1A4421,M1A4491,M1A4511,M1A4521,M1A4591,M1A4611,M1A4621,M1A4691,M1A4711,M1A4721,M1A4791,M1A48X1,M1A49X1,M1A9XX1,2740,27400,27401,27402,27403,27410,27419,27481,27482
Gout		27489,2749,M10,M100,M1000,M1001,M10011,M10012,M10019,M1002,M10021,M10022,M10029,M1003,M10031,M10032,

	M10039,M1004,M10041,M10042,M10049,M1005,M10051,M10052,M10059,M1006,M10061,M10062,M10069,M1007,M10071,M10072,M10079,M1008,M1009,M101,M1010,M1011,M10111,M10112,M10119,M1012,M10121,M10122,M10129,M1013,M10131,M10132,M10139,M1014,M10141,M10142,M10149,M1015,M10151,M10152,M10159,M1016,M10161,M10162,M10169,M1017,M10171,M10172,M10179,M1018,M1019,M102,M1020,M1021,M10211,M10212,M10219,M1022,M10221,M10222,M10229,M1023,M10231,M10232,M10239,M1024,M10241,M10242,M10249,M1025,M10251,M10252,M10259,M1026,M10261,M10262,M10269,M1027,M10271,M10272,M10279,M1028,M1029,M103,M1030,M1031,M10311,M10312,M10319,M1032,M10321,M10322,M10329,M1033,M10331,M10332,M10339,M1034,M10341,M10342,M10349,M1035,M10351,M10352,M10359,M1036,M10361,M10362,M10369,M1037,M10371,M10372,M10379,M1038,M1039,M104,M1040,M1041,M10411,M10412,M10419,M1042,M10421,M10422,M10429,M1043,M10431,M10432,M10439,M1044,M10441,M10442,M10449,M1045,M10451,M10452,M10459,M1046,M10461,M10462,M10469,M1047,M10471,M10472,M10479,M1048,M1049,M109,M1A,M1A0,M1A00,M1A00X0,M1A00X1,M1A01,M1A011,M1A0110,M1A0111,M1A012,M1A0120,M1A0121,M1A019,M1A0190,M1A0191,M1A02,M1A021,M1A0210,M1A0211,M1A022,M1A0220,M1A0221,M1A029,M1A0290,M1A0291,M1A03,M1A031,M1A0310,M1A0311,M1A032,M1A0320,M1A0321,M1A039,M1A0390,M1A0391,M1A04,M1A041,M1A0410,M1A0411,M1A042,M1A0420,M1A0421,M1A049,M1A0490,M1A0491,M1A05,M1A051,M1A0510,M1A0511,M1A052,M1A0520,M1A0521,M1A059,M1A0590,M1A0591,M1A06,M1A061,M1A0610,M1A0611,M1A062,M1A0620,M1A0621,M1A069,M1A0690,M1A0691,M1A07,M1A071,M1A0710,M1A0711,M1A072,M1A0720,M1A0721,M1A079,M1A0790,M1A0791,M1A08,M1A08X0,M1A08X1,M1A09,M1A09X0,M1A09X1,M1A1,M1A10,M1A10X0,M1A10X1,M1A11,M1A111,M1A1110,M1A1111,M1A112,M1A1120,M1A1121,M1A119,M1A1190,M1A1191,M1A12,M1A121,M1A1210,M1A1211,M1A122,M1A1220,M1A1221,M1A129,M1A1290,M1A1291,M1A13,M1A131,M1A1310,M1A1311,M1A132,M1A1320,M1A1321,M1A139,M1A1390,M1A1391,M1A14,M1A141,M1A1410,M1A1411,M1A142,M1A1420,M1A1421,M1A149,M1A1490,M1A1491,M1A15,M1A151,M1A1510,M1A1511,M1A152,M1A1520,M1A1521,M1A159,M1A1590,M1A1591,M1A16,M1A161,M1A1610,M1A1611,M1A162,M1A1620,M1A1621,M1A169,M1A1690,M1A1691,M1A17,M1A171,M1A1710,M1A1711,M1A172,M1A1720,M1A1721,M1A179,M1A1790,M1A1791,M1A18,M1A18X0,M1A18X1,M1A19,M1A19X0,M1A19X1,M1A2,M1A20,M1A20X0,M1A20X1,M1A21,M1A211,M1A2110,M1A2111,M1A212,M1A2120,M1A2121,M1A219,M1A2190,M1A2191,M1A22,M1A221,M1A2210,M1A2211,M1A222,M1A2220,M1A2221,M1A229,M1A2290,M1A2291,M1A23,M1A231,M1A2310,M1A2311,M1A232,M1A2320,M1A2321,M1A239,M1A2390,M1A2391,M1A24,M1A241,M1A2410,M1A2411,M1A242,M1A2420,M1A2421,M1A249,M1A2490,M1A2491,M1A25,M1A251,M1A2510,M1A2511,M1A252,M1A2520,M1A2521,M1A259,M1A2590,M1A2591,M1A26,M1A261,M1A2610,M1A2611,M1A262,M1A2620,M1A2621,M1A269,M1A2690,M1A2691,M1A27,M1A271,M1A2710,M1A2711,M1A272,M1A2720,M1A2721,M1A279,M1A2790,M1A2791,M1A28,M1A28X0,M1A28X1,M1A29,M1A29X0,M1A29X1,M1A3,M1A30,M1A30X0,M1A30X1,M1A31,M1A311,M1A3110,M1A3111,M1A312,M1A3120,M1A3121,M1A319,M1A3190,M1A3191,M1A32,M1A321,M1A3210,M1A3211,M1A322,M1A3220,M1A3221,M1A329,M1A3290,M1A3291,M1A33,M1A331,M1A3310,M1A3311,M1A332,M1A3320,M1A3321,M1A339,M1A3390,M1A3391,M1A34,M1A341,M1A3410,M1A3411,M1A342,M1A3420,M1A3421,M1A349,M1A3490,M1A3491,M1A35,M1A351,M1A3510,M1A3511,M1A352,M1A3520,M1A3521,M1A359,M1A3590,M1A3591,M1A36,M1A361,M1A3610,M1A3611,M1A362,M1A3620,M1A3621,M1A369,M1A3690,M1A3691,M1A37,M1A371,M1A3710,M1A3711,M1A372,M1A3720,M1A3721,M1A379,M1A3790,M1A3791,M1A38,M1A38X0,M1A38X1,M1A39,M1A39X0,M1A39X1,M1A4,M1A40,M1A40X0,M1A40X1,M1A41,M1A411,M1A4110,M1A4111,M1A412,M1A4120,M1A4121,M1A419,M1A4190,M1A4191,M1A42,M1A421,M1A4210,M1A4211,M1A422,M1A4220,M1A4221,M1A429,M1A4290,M1A4291,M1A43,M1A431,M1A4310,M1A4311,M1A432,M1A4320,M1A4321,M1A439,M1A4390,M1A4391,M1A44,M1A441,M1A4410,M1A4411,M1A442,M1A4420,M1A4421,M1A449,M1A4490,M1A4491,M1A45,M1A451,M1A4510,M1A4511,M1A452,M1A4520,M1A4521,M1A459,M1A4590,M1A4591,M1A46,M1A461,M1A4610,M1A4611,M1A462,M1A4620,M1A4621,M1A469,M1A4690,M1A4691,M1A47,M1A471,M1A4710,M1A4711,M1A472,M1A4720,M1A4721,M1A479,M1A4790,M1A4791,M1A48,M1A48X0,M1A48X1,M1A49,M1A49X0,M1A49X1,M1A9,M1A9XX0,M1A9XX1,V775
Lupus erythematosus	37334,6954,7100,H0112,H01121,H01122,H01123,H01124,H01125,H01126,H01129,L93,L930,L931,L932,M32,M320,M321,M3210,M3211,M3212,M3213,M3214,M3215,M3219,M328,M329,
Sjögren's syndrome	M3500,M3501,M3502,M3503,M3504,M3509,
Systemic sclerosis	5172,7101,M34,M340,M342,M348,M3481,M3482,M3483,M3489,M349,
Dermatomyositis	7103,M330,M3300,M3301,M3302,M3303,M3309,M331,M3310,M3311,M3312,M3313,M3319
Antiphospholipid antibody syndrome	28653,D68312,D6861
Polyarteritis nodosa	4460,M30,M300,M308
Wegener's granulomatosis	4464,M313,M3130,M3131
Giant cell arteritis	4465,M315,M316
Rheumatoid vasculitis	M052,M0520,M0521,M05211,M05212,M05219,M0522,M05221,M05222,M05229,M0523,M05231,M05232,M05239,M0524,M05241,M05242,M05249,M0525,M05251,M05252,M05259,M0526,M05261,M05262,M05269,M0527,M05271,M05272,M05279,M0529
Behcet's syndrome	1361,71120,71121,71122,71123,71124,71125,71126,71127,71128,71129,M352
Other connective tissue disorders	7109
Arthritis	00323,05671,09850,71100,71101,71102,71103,71104,71105,71106,71107,71108,71109,71190,71191,71192,71193,71194,71195,71196,71197,71198,71199,71620,71621,71622,71623,71624,71625,71626,71627,71628,71629,71630,71631,71632,71633,71634,71635,71636,71637,71638,71639,71650,71651,71652,71653,71654,71655,71656,71657,71658,71659,71660,71661,71662,71663,71664,71665,71666,71667,71668,7202,A0104,A0223,A1802,A3983,A3984,A5442,A6923,,
	B0682,B2685,B4282,E7881,E790,G980,L4052,M00,M000,M0000,M0001,M00011,M00012,,M00019,M0002,M00021,M00022,M00029,M0003,M00031,M00032,M00039,M0004,M00041,M00042,M00049,M0005,M00051,M00052,M00059,M0006,M00061,M00062,M00069,M0007,M00071,M00072,M00079,M0008,M0009,M001,M0010,M0011,M00111,M00112,M00119,M0012,M00121,M00122,M00129,M0013,M00131,M00132,M00139,M0014,M00141,M00142,M00149,M0015,M00151,M00152,M00159,M0016,M00161,M00162,M00169,M0017,M00171,M00172,M00179,M0018,M0019,M002,M0020,M0021,M00211,M00212,M00219,M0022,M00221,M00222,M00229,M0023,M00231,M00232,M00239,M0024,M00241,M00242,M00249,M0025,M00251,M00252,M00259,M0026,M00261,M00262,M00269,M0027,M00271,M00272,M00279,M0028,M0029,M008,M0080,M0081,M00811,M00812,M00819,M0082,M00821,M00822,M00829,M0083,M00831,M00832,M00839,M0084,M00841,M00842,M00849,M0085,M00851,M00852,M00859,M0086,M00861,M00862,M00869,M0087,M00871,M00872,M00879,M0088,M0089,M009,M08,M088,M0880,M0881,M08811,M08812,M08819,M0882,M08821,M08822,M08829,M0883,M08831,M08832,M08839,M0884,M08841,M08842,M08849,M0885,M08851,M08852,M08859,M0886,M08861,M08862,M08869,M0887,M08871,M08872,M08879,M0888,M0889,M089,M0890,M0891,M08911,M08912,M08919,M0892,M08921,M08922,M08929,M0893,M08931,M08932,M08939,M0894,M08941,M08942,M08949,M0895,M08951,M08952,M08959,M0896,M08961,M08962,M08969,M0897,M08971,M08972,M08979,M0898,M0899,M13,M130,M131,M1310,M1311,M13111,M13112,M13119,M1312,M13121,M13122,M13129,M1313,M13131,M13132,M13139,M1314,M13141,M13142,M13149,M1315,M13151,M13152,M13159,M1316,M13161,M13162,M13169,M1317,M13171,M13172,M13179,M138,M1380,M1381,M13811,M13812,M13819,M1382,M13821,M13822,M13829,M1383,M13831,M13832,M13839,M1384,M13841,M13842,M13849,M1385,M13851,M13852,M13859,M1386,M13861,M13862,M13869,M1387,M13871,M13872,M13879,M1388,M1389,M15,M150,M153,M154,M158,M159,M16,M160,M161,M1610,M1611,M1612,M162,M163,M1630,M1631,M1632,M164,M165,M1650,M1651,M1652,M166,M167,M169,M17,M170,M171,M1710,M1711,M1712,M172,M173,M1730,M1731,M1732,M174,M175,M179,M18,M180,M181,M1810,M1811,M1812,M182,M183,M1830,M1831,M1832,M184,M185,M1850,M1851,M1852,M189,M19,M190,M1901,M19011,M19012,M19019,M1902,M19021,M19022,M19029,M1903,M19031,M19032,M19039,M1904,M19041,M19042,M19049,M1907,M19071,M19072,M19079,M191,M1911,M19111,M19112,M19119,M1912,M19121,M19122,M19129,M1913,M19131,M19132,M19139,M1914,M19141,M19142,M19149,M1917,M19171,M19172,M19179,M192,M1921,M19211,M19212,M19219,M1922,M19221,M19222,M19229,M1923,M19231,M19232,M19239,M1924,M19241,M19242,M19249,M1927,M19271,M19272,M19279,M199,M1990,M1991,M1992,M1993,M461,M7720,M7721,M7722,V134,V177,V821,Z826,Z8261

eTable 5. Summary of Variables and Percentage of Missing Data in the GoutRe and MIMIC-IV Cohorts

Variable name		Data type	Labels	GoutRe cohort	MIMIC-IV cohort
**Demographics**	Age	Numeral	Year	0.64%	0%
	Sex	Factor	1.Male,2.Female	0.64%	0%
	Weight changed	Factor	1.Yes,2.No	2.42%	10.90%
**Lifestyle factors**	Smoking history	Factor	1.Yes,2.No	2.45%	0%
	Drinking history	Factor	1.Yes,2.No	2.42%	0%
**Laboratory**	UA	Factor	<6,<br>6.0-6.9,<br>7.0-7.9,<br>8.0-8.9,<br>9.0-9.9,<br>＞10<br>mg/dL	2.24%	74.80%
	eGFR	Factor	⩾90,<br>60-89,<br>45-59,<br>30-44,<br>15-29,<br>＜15<br>mL/min/1.73m²	2.87%	0%
**Physical examination**	Tophus	Factor	1.Yes,2.No	7.50%	0%
**Comorbidities**	Hypertension	Factor	1.Yes,2.No	1.47%	0%
	Diabetes	Factor	1.Yes,2.No	1.47%	0%
	Cardiovascular disease	Factor	1.Yes,2.No	1.47%	0%
	Heart failure	Factor	1.Yes,2.No	1.47%	0%
	Stroke	Factor	1.Yes,2.No	1.47%	0%
	Dyslipidemia	Factor	1.Yes,2.No	0.75%	0%
	Fatty liver	Factor	1.Yes,2.No	1.47%	0%
	Renal disease	Factor	1.Yes,2.No	1.47%	0%
	Thyroid disease	Factor	1.Yes,2.No	2.11%	0%
	Cancer	Factor	1.Yes,2.No	1.47%	0%
	Stones	Factor	1.Yes,2.No	1.47%	0%
	MetS	Factor	1.Yes,2.No	0%	0%
**Medication**	Urate-lowering therapy[a]	Factor	1.Yes,2.No	7.70%	0%
	Sodium bicarbonate	Factor	1.Yes,2.No	7.70%	0%
	GCs	Factor	1.Yes,2.No	7.56%	0%
	SGLT2 inhibitors	Factor	1.Yes,2.No	7.70%	0%
	CCB	Factor	1.Yes,2.No	7.70%	0%
	Losartan	Factor	1.Yes,2.No	7.70%	0%
	NL-ARBs	Factor	1.Yes,2.No	7.70%	0%
	β-blocker	Factor	1.Yes,2.No	7.70%	0%
	Diuretic	Factor	1.Yes,2.No	7.70%	0%
	Low-dose ASA[b]	Factor	1.Yes,2.No	7.70%	0%
	Statins	Factor	1.Yes,2.No	7.70%	0%
	Fenofibrate	Factor	1.Yes,2.No	7.70%	0%
	Mannitol	Factor	1.Yes,2.No	7.70%	0%

Abbreviations: UA, Uric acid; eGFR, Estimated glomerular filtration rate; MetS, Metabolic syndrome; GCs, Glucocorticoids; SGLT2 inhibitors, Sodium-glucose co-transporter 2 inhibitors; CCB, Calcium channel blockers; NL-ARBs, Non-losartan angiotensin II receptor blockers; β-blocker, Beta-blocker; ASA, Acetylsalicylic acid.

[a] Urate-lowering therapy included allopurinol, febuxostat, benzbromarone, while the MIMIC-IV cohort excludes Benzbromarone.

[b] Low dose refers to less than 100mg.

eTable 6. R environment and packages used for machine-learning and statistical analyses

Component	Description	Version	Source
**R**	Base environment	4.3.2	CRAN
**survival**	Cox regression / Fine–Gray competing-risk models	3.5-7	CRAN
**mice**	Multiple imputation (CART method)	3.16.0	CRAN
**readxl**	Import Excel files	1.4.3	CRAN
**writexl**	Export Excel files	1.4.2	CRAN
**randomForestSRC**	Random Survival Forest implementation	3.2.3	CRAN
**e1071**	Support Vector Machine (SVM) algorithms	1.7-14	CRAN
**xgboost**	Extreme Gradient Boosting (XGBoost)	1.7.6.1	CRAN
**riskRegression**	Model evaluation (AUC, C-index, Brier score)	2023.04.07	CRAN
**survIDINRI**	NRI / IDI for survival models	1.1-3	CRAN
**pROC**	ROC curve and DeLong test	1.18.0	CRAN
**rmda**	Decision-curve analysis (DCA)	1.6	CRAN
**Hmisc**	Data summary and utility functions	5.1-1	CRAN
**dplyr**	Data manipulation and pipelining	1.1.2	CRAN
**knitr**	Dynamic report generation in R	1.43	CRAN
**kableExtra**	Table formatting for publication	1.3.4	CRAN

eTable 7. Hyperparameters and optimization strategy

Model	Key hyperparameters	Search range / method	Final selected values	Validation strategy
**Cox**	–	–	–	Five imputed datasets, results pooled via Rubin's rule
**RSF**	ntree, mtry, nodesize, splitrule	Grid search (ntree 500–1500; nodesize 5–15; splitrule logrank/logrankscore)	ntree = 1000, mtry = √p, nodesize = 10, splitrule = logrank	10-fold CV within each imputed dataset
**SVM**	kernel, cost, gamma	Grid search (cost 0.1–10; gamma 0.001–0.1)	kernel = radial, cost = 1, gamma = 0.01	10-fold CV (mean AUC)
**XGBoost**	eta, max_depth, subsample, colsample_bytree	Random search (eta 0.01–0.3; max_depth 3–8; subsample 0.6–1.0)	eta = 0.05, max_depth = 6, subsample = 0.8, colsample_bytree = 0.7	10-fold CV (mean AUC)

Abbreviations: Hyperparameters were optimized using 10-fold cross-validation; the 1-SE rule selected parsimonious models.

eTable 8. Multivariate Cox Regression Analysis of Factors Associated with Inpatient Gout Recurrence in GoutRe and MIMIC-IV Cohorts

Variable	GoutRe cohort multifactor				MIMIC-IV cohort multifactor			
	HR	95%CI lower	95%CI upper	*P* value	HR	95%CI lower	95%CI upper	*P* value
**Demographic**								
Age	1.00	1.00	1.01	0.60	0.71	0.55	0.91	0.01
Sex	1.10	0.94	1.30	0.24	0.87	0.67	1.13	0.30
Weight changed	1.22	1.05	1.42	<0.01	0.77	0.56	1.05	0.12
**Gout recurrence**								
NLR-SD	1.00	0.96	1.05	0.97	0.83	0.71	0.97	0.02
NLR	2.05	1.83	2.30	<0.01	2.84	2.08	3.87	<0.01
**Lifestyle factors**								
Smoking history	1.00	0.89	1.12	0.89	1.05	0.60	1.84	0.87
Drinking history	1.13	1.01	1.27	0.04	1.17	0.84	1.63	0.36
**Laboratory**								
UA	1.18	1.14	1.21	<0.01	1.11	1.04	1.18	0.02
eGFR	0.96	0.92	1.01	0.11	1.17	1.06	1.28	<0.01
**Tophus**	1.74	1.48	2.04	<0.01	1.53	0.74	3.16	0.27
**Comorbidities**								
Hypertension	1.10	0.97	1.25	0.13	0.99	0.76	1.27	0.91
Diabetes	1.07	0.94	1.22	0.31	1.09	0.86	1.38	0.47
Cardiovascular disease	1.01	0.89	1.16	0.86	1.02	0.79	1.32	0.88
Heart failure	1.00	0.82	1.22	0.95	1.30	1.00	1.70	0.06
Stroke	1.32	1.16	1.49	<0.01	1.10	0.78	1.57	0.59
Dyslipidemia	1.09	0.97	1.22	0.14	0.89	0.71	1.13	0.35
Fatty liver	1.19	1.05	1.35	0.01	0.98	0.36	2.69	0.87
Renal disease	0.91	0.79	1.05	0.20	1.30	0.94	1.80	0.12
Thyroid disease	1.03	0.87	1.23	0.73	1.00	0.74	1.35	0.92
Cancer	0.93	0.82	1.06	0.30	0.82	0.63	1.08	0.154
Stones	0.95	0.85	1.05	0.29	0.75	0.43	1.33	0.33
MetS	0.93	0.77	1.13	0.47	NA	NA	NA	NA
**Medication**								
Urate-lowering therapy[a]	1.14	1.02	1.27	0.02	0.44	0.35	0.56	<0.01
Sodium bicarbonate	1.08	0.96	1.21	0.21	0.78	0.38	1.60	0.50
GCs	0.46	0.40	0.54	<0.01	0.36	0.18	0.75	<0.01
SGLT2 inhibitors	0.99	0.78	1.25	0.91	NA	NA	NA	NA
CCB	0.98	0.87	1.10	0.72	0.34	0.15	0.78	0.01
Losartan	0.87	0.70	1.08	0.20	0.16	0.02	1.18	0.07
NL-ARBs	0.82	0.71	0.94	0.00	NA	NA	NA	NA
β-blocker	0.91	0.80	1.02	0.12	0.36	0.20	0.64	<0.01
Diuretic	1.00	0.88	1.14	0.93	0.12	0.08	0.19	<0.01
Low-dose Aspirin(ASA)[b]	1.09	0.95	1.26	0.22	0.75	0.38	1.49	0.41
Statins	1.08	0.96	1.22	0.19	0.35	0.14	0.86	0.02
Fenofibrate	1.04	0.74	1.48	0.81	NA	NA	NA	NA
Mannitol	0.88	0.65	1.18	0.38	NA	NA	NA	NA

Abbreviations: NLR-SD, Neutrophil-Lymphocyte Ratio-Standard Deviation; NLR, Neutrophil-Lymphocyte Ratio; UA, Uric acid; eGFR, Estimated glomerular filtration rate; MetS, Metabolic syndrome; GCs, Glucocorticoids; SGLT2 inhibitors, Sodium-glucose co-transporter 2 inhibitors; CCB, Calcium channel blockers; NL-ARBs, Non-losartan angiotensin II receptor blockers; β-blocker, Beta-blocker; ASA, Acetylsalicylic acid.

[a] Urate-lowering therapy included allopurinol, febuxostat, benzbromarone, while the MIMIC-IV cohort excludes Benzbromarone.

[b] Low dose refers to less than 100mg.

eTable 9. Multivariate-Adjusted Hazard Ratios (95% CI) of NLR for Gout Inpatient Gout Recurrence With Normal Serum Uric Acid

Characteristic	Model 1[a]		Model 2[b]		Model 3[c]	
	HR (95% CI)	P value	HR (95% CI)	P value	HR (95% CI)	P value
**GoutRe cohort**						
NLR						
Lower	Ref	Ref	Ref	Ref	Ref	Ref
Higher	2.34 (1.92,2.86)	<0.01	2.43 (1.96,3.01)	<0.01	2.38(1.91,2.96)	<0.01
NLR-SD[d]	1.12(1.07,1.17)	<0.01	1.05(0.97,1.12)	0.22	1.06(0.99,1.14)	0.10
UA[e]						
<6	Ref	Ref	Ref	Ref	Ref	Ref
6.0-6.9	1.23 (1.02,1.49)	0.03	1.27(1.05,1.53)	0.01	1.30(1.07,1.57)	0.01
eGFR[f]						
≥90	Ref	Ref	Ref	Ref	Ref	Ref
60-89	1.07(0.85,1.34)	0.60	1.02 (0.81,1.29)	0.85	1.08(0.84,1.39)	0.58
45-59	1.17 (0.88,1.57)	0.29	1.13(0.84,1.51)	0.43	1.14(0.82,1.58)	0.47
30-44	1.21(0.87,1.70)	0.28	1.12(0.80,1.57)	0.53	1.09(0.73,1.63)	0.67
15-29	1.01(0.67,1.52)	0.78	0.85 (0.56,1.29)	0.47	0.74(0.44,1.22)	0.25
<15	0.78(0.56,1.08)	0.14	0.70(0.50,0.97)	0.03	0.62(0.40,0.97)	0.04
Tophus						
No	Ref	Ref	Ref	Ref	Ref	Ref
Yes	2.37 (1.82,3.10)	<0.01	2.45(1.87,3.20)	<0.01	2.43(1.84,3.21)	<0.01
**MIMIC-IV cohort**						
NLR						
Lower	Ref	Ref	Ref	Ref	Ref	Ref
Higher	1.72 (0.77,3.89)	0.19	2.33(0.96,5.63)	0.06	3.28(1.12,9.63)	0.03
NLR-SD[d]	0.94 (0.69,1.28)	0.71	0.84(0.57,1.24)	0.39	0.62(0.37,1.05)	0.07
UA[e]						
<6	Ref	Ref	Ref	Ref	Ref	Ref
6.0-6.9	0.82(0.43,1.57)	0.55	0.88(0.46,1.69)	0.70	1.16(0.56,2.40)	0.68
eGFR[f]						
≥90	Ref	Ref	Ref	Ref	Ref	Ref
60-89	0.71(0.30,1.64)	0.42	0.65(0.28,1.51)	0.32	1.58(0.49,5.10)	0.45
45-59	0.51 (0.16,1.64)	0.26	0.46(0.14,1.52)	0.20	0.59(0.12,2.92)	0.52
30-44	0.99 (0.41,2.39)	0.98	0.74(0.29,1.89)	0.53	1.78(0.47,6.69)	0.40
15-29	0.48 (0.13,1.76)	0.27	0.38(0.10,1.42)	0.15	2.16(0.29,16.01)	0.46
<15	0.63 (0.20,2.02)	0.43	0.53(0.16,1.73)	0.30	1.60(0.25,10.39)	0.63
Tophus						
No	Ref	Ref	Ref	Ref	Ref	Ref
Yes	2.92(0.90,9.50)	0.07	3.49(1.04,11.73)	0.04	3.81(0.87,16.75)	0.08

Abbreviations: HR, Hazard Ratio; NLR, Neutrophil-Lymphocyte Ratio; UA, Uric acid; eGFR, Estimated glomerular filtration rate.

[a] Model 1 is the unadjusted model.

[b] Model 2 is adjusted for only the remaining 3 factors of NLR, UA, eGFR, Tophus, in addition to the factors being analyzed. For example, UA, eGFR, and Tophus are adjusted when NLR is analyzed.

[c] Model 3 is adjusted for model 2 covariates plus multivariable,including age, Sex, Smoking history, Drinking history, Weight changed, Hypertension, Diabetes, Cardiovascular diseased, Heart failure, Stroke, Dyslipidemia, Fatty liver, Renal disease, Thyroid disease, Cancer, Stones, MetS, Urate-lowering therapy, Sodium bicarbonate, GCs, SGLT2 inhibitors, CCB, Losartan, NL-ARBs, β-blockers, Diuretic, Low-dose Aspirin, Statins, Fenofibrate, Mannitol.

[d] Per 1SD of Neutrophil-Lymphocyte Ratio.

[e] Per 1mg/dL of Uric acid.

[f] Per 1mL/min/1.73m^2 of Estimated glomerular filtration rate.

eTable 10. Multivariate-Adjusted Hazard Ratios (95% CI) of NLR for Inpatient Gout Recurrence Without Tophus

Characteristic	Model 1[a]		Model 2[b]		Model 3[c]	
	HR (95% CI)	P value	HR (95% CI)	P value	HR (95% CI)	P value
**GoutRe cohort**						
NLR						
Lower	Ref	Ref	Ref	Ref	Ref	Ref
Higher	1.54(1.38,1.71)	<0.01	1.67(1.48,1.88)	<0.01	1.76(1.56,1.98)	<0.01
NLR-SD[d]	1.04(1.00,1.07)	0.03	0.98 (0.93,1.03)	0.50	1.02(0.97,1.07)	0.54
UA[e]						
<6	Ref	Ref	Ref	Ref	Ref	Ref
6.0-6.9	1.31(1.07,1.59)	0.01	1.35(1.10,1.64)	<0.01	1.35(1.10,1.64)	<0.01
7.0-7.9	1.57(1.30,1.89)	＜0.01	1.65 (1.37,1.99)	＜0.01	1.69(1.40,2.03)	＜0.01
8.0-8.9	1.63(1.36,1.96)	＜0.01	1.76(1.47,2.11)	＜0.01	1.78(1.48,2.14)	＜0.01
9.0-9.9	1.81(1.49,2.19)	＜0.01	1.93 (1.59,2.34)	＜0.01	1.95(1.60,2.37)	＜0.01
＞10	2.29 (1.94,2.70)	＜0.01	2.42(2.04,2.86)	＜0.01	2.40 (2.02,2.86)	＜0.01
eGFR[f]						
≥90	Ref	Ref	Ref	Ref	Ref	Ref
60-89	1.11(0.96,1.30)	0.16	1.04 (0.90,1.21)	0.59	1.03(0.88,1.21)	0.70
45-59	1.21 (1.01,1.45)	0.04	1.03 (0.86,1.24)	0.73	1.01(0.83,1.23)	0.91
30-44	1.21(1.00,1.46)	0.06	0.95(0.78,1.15)	0.58	0.93(0.74,1.16)	0.51
15-29	1.17 (0.95,1.44)	0.14	0.84(0.68,1.04)	0.11	0.87(0.67,1.13)	0.30
<15	1.11(0.92,1.33)	0.28	0.84 (0. 70,1.02)	0.08	0.86(0.67,1.12)	0.26
**MIMIC-IV cohort**						
NLR						
Lower	Ref	Ref	Ref	Ref	Ref	Ref
Higher	1.69(1.27,2.25)	<0.01	2.14 (1.58,2.92)	<0.01	2.94(2.15,4.03)	<0.01
NLR-SD[d]	0.90(0.79,1.02)	0.10	0.75(0.63,0.89)	<0.01	0.83(0.71,0.97)	0.02
UA[e]						
<6	Ref	Ref	Ref	Ref	Ref	Ref
6.0-6.9	0.92 (0.48,1.78)	0.81	0.90 (0.47,1.75)	0.77	1.16(0.58,2.33)	0.67
7.0-7.9	1.75(0.98,3.13)	0.06	1.87(1.04,3.36)	0.04	1.57(0.85,2.92)	0.16
8.0-8.9	1.09(0.55,2.15)	0.81	1.15(0.58,2.29)	0.70	1.03(0.50,2.13)	0.89
9.0-9.9	1.07 (0.49,2.36)	0.86	1.16(0.52,2.58)	0.72	0.91(0.38,2.14)	0.82
＞10	2.49 (1.49,4.18)	<0.01	2.47 (1.45,4.20)	<0.01	2.26(1.24,4.10)	0.01
eGFR[f]						
≥90	Ref	Ref	Ref	Ref	Ref	Ref
60-89	0.77 (0.53,1.14)	0.19	0.72 (0.49,1.06)	0.10	0.87 (0.58,1.31)	0.51
45-59	1.16(0.75,1.78)	0.51	0.94 (0.60,1.47)	0.79	1.83(1.12,3.01)	0.02
30-44	1.49 (1.01,2.19)	0.04	1.17(0.78,1.74)	0.46	2.34 (1.42,3.88)	<0.01
15-29	1.44(0.97,2.13)	0.07	1.07(0.71,1.61)	0.76	2.31(1.36,3.92)	<0.01
<15	1.32(0.85,2.05)	0.22	1.00(0.63,1.59)	0.94	1.59(0.90,2.80)	0.11

Abbreviations: HR, Hazard Ratio; NLR, Neutrophil-Lymphocyte Ratio; UA, Uric acid; eGFR, Estimated glomerular filtration rate.

[a] Model 1 is the unadjusted model.

[b] Model 2 is adjusted for only the remaining 3 factors of NLR, UA, eGFR, Tophus, in addition to the factors being analyzed. For example, UA, eGFR, and Tophus are adjusted when NLR is analyzed.

[c] Model 3 is adjusted for model 2 covariates plus multivariable,including age, Sex, Smoking history, Drinking history, Weight changed, Hypertension, Diabetes, Cardiovascular diseased, Heart failure, Stroke, Dyslipidemia, Fatty liver, Renal disease, Thyroid disease, Cancer, Stones, MetS, Urate-lowering therapy, Sodium bicarbonate, GCs, SGLT2 inhibitors, CCB, Losartan, NL-ARBs, β-blockers, Diuretic, Low-dose Aspirin, Statins, Fenofibrate, Mannitol.

[d] Per 1SD of Neutrophil-Lymphocyte Ratio.

[e] Per 1mg/dL of Uric acid.

[f] Per 1mL/min/1.73m^2 of Estimated glomerular filtration rate.

eTable 11. Multivariate-Adjusted Hazard Ratios (95% CI) of NLR for Inpatient Gout Recurrence Without Tophus and Normal Serum Uric Acid

Characteristic		Model 1[a]		Model 2[b]		Model 3[c]	
		HR (95% CI)	P value	HR (95% CI)	P value	HR (95% CI)	P value
**GoutRe cohort**							
NLR							
	Lower	Ref	Ref	Ref	Ref	Ref	Ref
	Higher	1.53(1.23,1.89)	<0.01	1.65(1.30,2.09)	<0.01	1.59(1.24,2.03)	<0.01
NLR-SD[d]		1,06 (0.99,1.14)	0.10	1.01(0.93,1.11)	0.80	1.05(0.95,1.16)	0.31
UAe							
	<6	Ref	Ref	Ref	Ref	Ref	Ref
	6.0-6.9	1.31(1.07,1.60)	0.01	1.36 (1.12,1.67)	<0.01	1.37(1.11,1.68)	<0.01
eGFR[f]							
	≥90	Ref	Ref	Ref	Ref	Ref	Ref
	60-89	1.01(0.79,1.30)	0.93	0.96(0.75,1.24)	0.78	1.03(0.78,1.37)	0.82
	45-59	0.98(0.71,1.36)	0.91	0.88(0.64,1.23)	0.47	0.97(0.67,1.49)	0.86
	30-44	0.98(0.67,1.44)	0.93	0.85 (0.58,1.25)	0.41	0.96(0.62,1.50)	0.86
	15-29	0.88(0.56,1.40)	0.59	0.78(0.49,1.23)	0.28	0.81(0.46,1.44)	0.48
	<15	0.60(0.42,0.88)	0.01	0.53(0.37,0.78)	<0.01	0.57(0.35,0.95)	0.03
**MIMIC-IV cohort**							
NLR[d]							
	Lower	Ref	Ref	Ref	Ref	Ref	Ref
	Higher	1.51(0.67,3.42)	0.32	1.98(0.81,4.82)	0.13	2.32(0.79,6.83)	0.13
NLR-SD		0.92(0.66,1.29)	0.64	0.84(0.56,1.26)	0.39	0.69(0.41,1.16)	0.16
UA[e]							
	<6	Ref	Ref	Ref	Ref	Ref	Ref
	6.0-6.9	0.92(0.47,1.78)	0.80	0.95(0.49,1.84)	0.87	1.64(0.76,3.54)	0.22
eGFR[f]							
	≥90	Ref	Ref	Ref	Ref	Ref	Ref
	60-89	0.71(0.31,1.64)	0.42	0.67(0.29,1.56)	0.35	1.58(0.48,5.18)	0.45
	45-59	0.53(0.17,1.69)	0.28	0.50 (0.16,1.63)	0.25	1.04(0.23,4.80)	0.93
	30-44	0.85(0.33,2.15)	0.73	0.76(0.29,1.96)	0.57	2.46(0.51,11.99)	0.30
	15-29	0.33(0.07,1.49)	0.15	0.30(0.06,1.32)	0.11	2.27(0.23,22.04)	0.50
	<15	0.62(0.19,1.99)	0.42	0.54(0.17,1.76)	0.31	2.13 (0.29,15. 50)	0.47

Abbreviations: HR, Hazard Ratio; NLR, Neutrophil-Lymphocyte Ratio; UA, Uric acid; eGFR, Estimated glomerular filtration rate.

[a] Model 1 is the unadjusted model.

[b] Model 2 is adjusted for only the remaining 3 factors of NLR, UA, eGFR, Tophus, in addition to the factors being analyzed. For example, UA, eGFR, and Tophus are adjusted when NLR is analyzed.

[c] Model 3 is adjusted for model 2 covariates plus multivariable,including age, Sex , Smoking history, Drinking history, Weight changed, Hypertension, Diabetes, Cardiovascular diseased, Heart failure, Stroke, Dyslipidemia, Fatty liver, Renal disease, Thyroid disease, Cancer, Stones, MetS, Urate-lowering therapy, Sodium bicarbonate, GCs, SGLT2 inhibitors, CCB, Losartan, NL-ARBs, β-blockers, Diuretic, Low-dose Aspirin, Statins, Fenofibrate, Mannitol.

[d] Per 1SD of Neutrophil-Lymphocyte Ratio.

[e] Per 1mg/dL of Uric acid.

[f] Per $1mL/min/1.73m^2$ of Estimated glomerular filtration rate.

eTable 12. Multivariate-Adjusted Hazard Ratios (95% CI) of NLR for Inpatient Gout Recurrence With Urate-lowering Therapy

Characteristic		Model 1[a]		Model 2[b]		Model 3[c]	
		HR (95% CI)	P value	HR (95% CI)	P value	HR (95% CI)	P value
**GoutRe cohort**							
NLR							
	Lower	Ref	Ref	Ref	Ref	Ref	Ref
	Higher	1.57(1.31,1.87)	<0.01	1.61(1.31,1.98)	<0.01	1.74(1.40,2.17)	<0.01
NLR-SD[d]		1.10(1.03,1.17)	0.30	1.01(0.93,1.10)	0.81	1.00(0.91,1.09)	0.92
UA[e]							
	<6	Ref	Ref	Ref	Ref	Ref	Ref
	6.0-6.9	1.15(0.78,1.69)	0.47	1.14(0.77,1.67)	0.51	1.20 (0.81,1.78)	0.36
	7.0-7.9	1.81(1.28,2.56)	<0.01	1.85(1.31,1.31)	<0.01	1.92(1.35,2.72)	＜0.01
	8.0-8.9	1.61(1.16,2.23)	<0.01	1.70(1.23,2.35)	<0.01	1.79(1.29,2.50)	<0.01
	9.0-9.9	1.84(1.32,2.56)	＜0.01	1.83(2.35,2.55)	<0.01	1.99(1.42,2.80)	＜0.01
	＞10	2.38(1.78,3.17)	＜0.01	2.43(1.82,3.24)	<0.01	2.56(1.89,3.45)	＜0.01
eGFR[f]							
	≥90	Ref	Ref	Ref	Ref	Ref	Ref
	60-89	0.96(0.73,1.26)	0.75	0.88(0.67,1.16)	0.37	0.87 (0.66,1.17)	0.37
	45-59	1.02(0.75,1.39)	0.89	0.88(0.64,1.20)	0.41	0.98(0.70,1.37)	0.88
	30-44	1.08(0.78,1.49)	0.67	0.85(0.61,1.19)	0.35	0.97(0.66,1.43)	0.85
	15-29	1.07(0.77,1.48)	0.68	0.80 (0.57,1.12)	0.20	1.00(0.67,1.50)	0.90
	<15	1.01(0.74,1.37)	0.94	0.75(0.55,1.03)	0.08	0.85(0.56,1.30)	0.46
Tophus							
	No	Ref	Ref	Ref	Ref	Ref	Ref
	Yes	1.87(1.47,2.36)	<0.01	1.71(1.35,2.17)	<0.01	1.75(1.37,2.24)	<0.01
**MIMIC-IV cohort**							
NLR[d]							
	Lower	Ref	Ref	Ref	Ref	Ref	Ref
	Higher	1.26(0.81,1.94)	0.31	1.66(1.02,2.69)	0.04	2.48(1.51,4.09)	<0.01
NLR-SD		0.86(0.68,1.08)	0.20	0.73(0.54,0.98)	0.04	0.81(0.60,1.08)	0.15
UA[e]							
	<6	Ref	Ref	Ref	Ref	Ref	Ref
	6.0-6.9	0.70(0.18,2.43)	0.54	0.68(0.19,2.51)	0.56	0.85(0.21,3.36)	0.81
	7.0-7.9	1.30(0.44,3.81)	0.63	1.37(0.45,4.12)	0.58	1.12(0.31,4.03)	0.87
	8.0-8.9	0.93(0.29,3.00)	0.91	1.00(0.30,3.29)	1.00	0.94(0.25,3.59)	0.91
	9.0-9.9	1.95(0.67,5.71)	0.22	1.89(0.63,5.68)	0.26	1.35(0.40,4.56)	0.63
	＞10	2.00(0.73,5.51)	0.18	1.79(0.63,5.09)	0.27	1.75(0.52,5.90)	0.37
eGFR[f]							
	≥90	Ref	Ref	Ref	Ref	Ref	Ref
	60-89	1.62 (0.71,3.71)	0.25	1.52(0.66,3.48)	0.32	1.35(0.57,3.23)	0.50
	45-59	2.32(0.96,5.60)	0.06	2.06(0.84,5.07)	0.13	1.84(0.70,4.85)	0.22
	30-44	2.65(1.14,6.18)	0.02	2.23(0.93,5.32)	0.08	2.15(0.78,5.90)	0.15
	15-29	2.25(0.95,5.32)	0.07	1.79(0.74,4.35)	0.21	1.84(0.66,5.14)	0.25
	<15	2.30(0.92,5.76)	0.08	0.92(0.74,4.93)	0.19	1.28(0.44,3.77)	0.66
Tophus							
	No	Ref	Ref	Ref	Ref	Ref	Ref
	Yes	4.07(1.66,9.98)	<0.01	3.77(1.53,9.30)	<0.01	4.41(1.65,11.79)	<0.01

Abbreviations: HR, Hazard Ratio; NLR, Neutrophil-Lymphocyte Ratio; UA, Uric acid; eGFR, Estimated glomerular filtration rate.

[a] Model 1 is the unadjusted model.

[b] Model 2 is adjusted for only the remaining 3 factors of NLR, UA, eGFR, Tophus, in addition to the factors being analyzed. For example, UA, eGFR, and Tophus are adjusted when NLR is analyzed.

[c] Model 3 is adjusted for model 2 covariates plus multivariable,including age, Sex , Smoking history, Drinking history, Weight changed, Hypertension, Diabetes, Cardiovascular diseased, Heart failure, Stroke, Dyslipidemia, Fatty liver, Renal disease, Thyroid disease, Cancer, Stones, MetS, Urate-lowering therapy, Sodium bicarbonate, GCs, SGLT2 inhibitors, CCB, Losartan, NL-ARBs, β-blockers, Diuretic, Low-dose Aspirin, Statins, Fenofibrate, Mannitol.

[d] Per 1SD of Neutrophil-Lymphocyte Ratio.

[e] Per 1mg/dL of Uric acid.

[f] Per 1mL/min/1.73m^2 of Estimated glomerular filtration rate.

eTable 13. Baseline Characteristics

	GoutRe cohort				MIMIC-IV cohort			
	**All (n=5584)**	**Low NLR (n=2848)**	**High NLR (n=2736)**	***P* value**	**All (n=2019)**	**Low NLR (n=582)**	**High NLR (n=1437)**	***P* value**
**Demographic**								
Age, mean(SD), y	62.72(14.66)	60.51 (14.58)	65 (14.40)	<0.01	67.98(12.93)	67.38(13.02)	68.23(12.89)	0.18
Race				<0.01				<0.01
Asian	5584(100%)	2848(100%)	2736(100%)		80 (3.98%)	20(3.43%)	60(4.20%)	
Black	0(0%)	0(0%)	0(0%)		346(17.22%)	157(26.97%)	189(13.24%)	
White	0(0%)	0(0%)	0(0%)		1464(72.87%)	368(63.23%)	1096(76.80%)	
Other[a]	0(0%)	0(0%)	0(0%)		119(3.02%)	37(6.36%)	82(5.74%)	
Sex								
Male	4816 (86.25%)	2433 (86.43%)	2383 (87.10%)	0.38	1516 (75.09%)	425(73.02%)	1091 (75.92%)	0.19
Female	732 (13.11%)	383 (13.57%)	349 (12.80%)		503 (24.91%)	157 (26.98%)	346 (24.08%)	
Weight changed[b]	580 (10.39%)	265 (9.30%)	315 (11.51%)	0.02	1521 (75.33%)	429 (73.71%)	1092 (75.99%)	0.40
**Gout recurrence**	1659 (29.71%)	573 (20.12%)	1086 (39.69%)	<0.01	320 (15.85%)	59 (10.14%)	261 (18.16%)	<0.01
**Lifestyle factors**								
Smoking history	2386 (42.73%)	1213 (42.59%)	1173 (42.87%)	0.85	91 (4.51%)	25 (4.3%)	66 (4.59%)	0.86
Drinking history	1862 (33.35%)	954 (33.5%)	908 (33.19%)	0.38	267 (13.22%)	72 (12.37%)	195 (13.57%)	0.52
**Laboratory**								
UA, mg/dL								
<6	1188 (21.28%)	546 (19.17%)	642 (23.46%)	<0.01	167 (8.27%)	47 (8.08%)	120 (8.35%)	0.91
6.0-6.9	776 (13.90%)	392 (13.76%)	384 (14.04%)	0.90	80 (3.96%)	15 (2.58%)	65 (4.52%)	0.06
7.0-7.9	903 (16.17%)	488 (17.13%)	415 (15.17%)	0.04	69 (3.42%)	22 (3.78%)	47 (3.27%)	0.66
8.0-8.9	879 (15.74%)	518 (18.19%)	361 (13.19%)	<0.01	60 (2.97%)	19 (3.26%)	41 (2.85%)	0.73
9.0-9.9	712 (12.75%)	367 (12.89%)	345 (12.61%)	0.79	45 (2.23%)	14 (2.41%)	31 (2.16%)	0.86
＞10	1001 (17.93%)	462 (16.22%)	539 (19.70%)	<0.01	91 (4.51%)	19 (3.26%)	72 (5.01%)	0.11
eGFR, mL/min/1.73m²								
≥90	1112 (19.91%)	756 (26.54%)	356 (13.01%)	<0.01	318 (15.75%)	84 (14.43%)	234 (16.28%)	0.33
60-89	1808 (32.38%)	1072 (37.64%)	736 (26.90%)	<0.01	656 (32.49%)	176 (30.24%)	480 (33.4%)	0.19
45-59	742 (13.29%)	347 (12.18%)	395 (14.44%)	0.03	254 (12.58%)	99 (17.01%)	155 (10.79%)	<0.01
30-44	595 (10.66%)	240 (8.43%)	355 (12.98%)	<0.01	302 (14.96%)	90 (15.46%)	212 (14.75%)	0.74
15-29	472 (8.45%)	155 (5.44%)	317 (11.59%)	<0.01	293 (14.51%)	75 (12.89%)	218 (15.17%)	0.21
<15	695 (12.45%)	172 (6.04%)	523 (19.12%)	<0.01	196 (9.71%)	58 (9.97%)	138 (9.6%)	0.87
**Tophus**[c]	327 (5.86%)	109 (3.83%)	218 (7.97%)	<0.01	23 (1.14%)	7 (1.2%)	16 (1.11%)	1.00
**Comorbidities**								
Hypertension	3360 (60.17%)	1601 (56.21%)	1759 (64.29%)	<0.01	780 (38.63%)	219 (37.63%)	561 (39.04%)	0.59
Diabetes	1736 (31.09%)	852 (29.92%)	884 (32.31%)	0.10	848 (42.00%)	233 (40.03%)	615 (42.80%)	0.28
Cardiovascular disease[d]	1281 (22.94%)	619 (21.73%)	662 (24.20%)	0.05	733 (36.31%)	195 (33.51%)	538 (37.44%)	0.11
Heart failure	337 (6.04%)	104 (3.65%)	233 (8.52%)	<0.01	771 (38.19%)	173 (29.73%)	598 (41.61%)	<0.01
Stroke[e]	912 (16.33%)	379 (13.31%)	533 (19.48%)	<0.01	213 (10.55%)	58 (9.97%)	155 (10.79%)	0.64
Dyslipidemia[f]	2675 (47.90%)	1416 (49.72%)	1259 (46.02%)	<0.01	1113 (55.13%)	326 (56.01%)	787 (54.77%)	0.64
Fatty liver	1352 (24.21%)	844 (29.63%)	508 (18.57%)	<0.01	21 (1.04%)	8 (1.37%)	13 (0.90%)	0.48

Renal disease[g]	1707 (30.57%)	619 (21.73%)	1088 (39.77%)	<0.01	1137 (56.32%)	328 (56.36%)	809 (56.30%)	1.00
Thyroid disease[h]	497 (8.90%)	275 (9.66%)	222 (8.11%)	0.03	375 (18.57%)	121 (20.79%)	254 (17.68%)	0.12
Cancer	1173 (21.01%)	636 (22.33%)	537 (19.63%)	0.01	576 (28.53%)	139 (23.88%)	437 (30.41%)	<0.01
Stones	2358 (42.23%)	1154 (40.52%)	1204 (44.01%)	<0.01	95 (4.71%)	24 (4.12%)	70 (4.94%)	0.50
MetS[i]	721 (12.91%)	355 (12.46%)	366 (13.38%)	0.33	6 (0.30%)	3 (0.52%)	3 (0.21%)	0.49
**Medication**								
Urate-lowering therapy[j]	1455 (26.06%)	719 (25.25%)	736 (26.90%)	0.03	188 (9.31%)	43 (7.39%)	145 (10.09%)	0.07
$NaHCO_3$	1399 (25.05%)	570 (20.01%)	829 (30.30%)	<0.01	74 (3.67%)	14 (2.41%)	60 (4.18%)	0.07
GCs	969 (17.35%)	387 (13.59%)	582 (21.27%)	<0.01	135 (6.69%)	38 (6.53%)	97 (6.75%)	0.93
SGLT2 inhibitors	288 (5.16%)	146 (5.13%)	142 (5.19%)	0.96	NA	NA	NA	NA
CCB	2081(37.27%)	911 (31.99%)	1170 (42.76%)	<0.01	114 (5.65%)	27 (4.64%)	87 (6.05%)	0.25
Losartan	289 (5.18%)	154 (5.41%)	135 (4.93%)	0.46	51 (2.53%)	17 (2.92%)	34 (2.37%)	0.57
NL-ARBs	972 (17.41%)	449 (15.77%)	523 (19.12%)	0.59	13 (0.64%)	2 (0.34%)	11 (0.77%)	0.37
β-blockers	1294 (23.17%)	529 (18.57%)	765 (27.96%)	<0.01	186 (9.21%)	34 (5.84%)	152 (10.58%)	<0.01
Diuretic[k]	930 (16.65%)	260 (9.13%)	670 (24.49%)	<0.01	586 (29.02%)	89 (15.29%)	497 (34.59%)	<0.01
Low-dose aspirin (ASA)[l]	956 (17.12%)	465 (16.33%)	491 (17.95%)	00.29	116 (5.75%)	22 (3.78%)	94 (6.54%)	0.02
Statins	1728 (30.95%)	827 (29.04%)	901 (32.93%)	0.41	117 (5.79%)	29 (4.98%)	88 (6.12%)	0.37
Fenofibrate	98 (1.76%)	66 (2.32%)	32 (1.17%)	<0.01	5 (0.25%)	1 (0.17%)	4 (0.28%)	1.00
Mannitol	133 (2.38%)	44 (1.54%)	89 (3.25%)	<0.01	NA	NA	NA	NA

Abbreviations: NLR, Neutrophil-Lymphocyte Ratio; UA, Uric Acid; eGFR, Estimated glomerular filtration rate; MetS, Metabolic Syndrome; NaHCO3, Sodium bicarbonate; GCs, Glucocorticoids; SGLT2 inhibitors, Sodium-glucose co-transporter two inhibitors; CCB, Calcium channel blockers; NL-ARBs, Non-losartan angiotensin II receptor blockers; β-blocker, Beta-blocker; ASA, Acetylsalicylic acid.

[a] Other regions include American Indian, Multiple, and others.

[b] Weight changed referred to any weight gain or loss in previous medical history compared with the last hospitalization or in the past six months.

[c] Tophus referred to deposit crystals of sodium urate (typically located in joints, ears, olecranon bursae, finger pads, and tendons), often accompanied by vascular coverage, reported positively on physical examination.

[d] Cardiovascular disease included heart diseases and coronary arteriovenous diseases caused by various causes, such as Angina pectoris (ICD-10:I20), Coronary atherosclerotic heart disease (ICD-10:I25.1), heart failure (ICD-10:I50), etc.

[e] Stroke included cerebral hemorrhage (ICD-10:I61) or cerebral infarction (ICD-10:I63).

[f] Dyslipidemia mainly referred to elevated or decreased levels of total cholesterol (TC), triglyceride (TG), low-density lipoprotein cholesterol (LDL-C), high-density lipoprotein cholesterol (HDL-C), and/(or) lipoprotein-α(LPα) in the plasma. (ICD-10:E78).

[g] Renal diseases were diagnosed with renal insufficiency (ICD-10:N18.0, N19), kidney failure (ICD-10:N17-N18), chronic kidney disease (ICD-10:N18.9), and uremia (ICD-10:N19.x01).

[h] Thyroid disease included hyperthyroidism (ICD-10:E05.901), hypothyroidism (ICD-10:E00-E03), thyroid nodules (ICD-10:E04-E05), thyroid cysts (ICD: E07.0), and thyroid tumors (ICD-10:C73), etc.

[i] MetS referred to metabolic disorders diagnosed simultaneously with hypertension, diabetes, and dyslipidemia.

[j] Urate-lowering therapy included allopurinol, febuxostat, and benzbromarone, while the MIMIC-IV cohort excludes Benzbromarone.

[k] Diuretic included thiazides and loop diuretics.

[l] Low dose refers to less than 100mg.

eTable 14. Improved Discrimination and Risk Reclassification for Inpatient Gout Recurrence With or Without NLR

Population	Model	C-statistic (95% Cl)	P value	NRI(95% CI)	P value	IDI (95% CI)	P value
**Inpatient gout recurrence**							
**GoutRe cohort**							
	Basic model	0.65(0.65,0.65)	Ref	Ref	Ref	Ref	Ref
	+NLR	0.68(0.68,0.68)	<0.01	0.00(-0.00,0.00)	0.35	0.52(0.34,0.76)	<0.01
**MIMIC-IV cohort**							
	Basic model	0.803(0.80,0.81)	Ref	Ref	Ref	Ref	Ref
	+NLR	0.81(0.81,0.82)	<0.01	0.00(-0.0,0.01)	0.29	0.71(0.12,2.10)	<0.01
**Inpatient gout recurrence with normal serum uric acid**							
**GoutRe cohort**							
	Basic model	0.63(0.63,0.63)	Ref	Ref	Ref	Ref	Ref
	+NLR	0.68(0.68,0.68)	<0.01	0.00(-0.00,0.00)	0.34	0.31(0.10,0.57)	<0.01
**MIMIC-IV cohort**							
	Basic model	0.69(0.69,0.69)	Ref	Ref	Ref	Ref	Ref
	+NLR	0.71(0.72,0.72)	0.07	0.00(-0.00,0.01)	0.10	0.37(0.08,0.80)	<0.01
**Cumulative incidence of inpatient gout recurrence among patients undergoing urate−lowering therapy**							
**GoutRe cohort**							
	Basic model	0.65(0.64,0.65)	Ref	Ref	Ref	Ref	Ref
	+NLR	0.67(0.67,0.67)	<0.01	0.00(-0.00,0.00)	0.28	0.69(0.28,1.20)	<0.01
**MIMIC-IV cohort**							
	Basic model	0.70(0.70,0.70)	Ref	Ref	Ref	Ref	Ref
	+NLR	0.72(0.72,0.72)	0.01	0.00(0.00,0.00)	0.02	0.32(0.03,0.77)	0.02

Inpatient Gout Recurrence

GoutRe：The basic model included UA, Tophus, Drinking History, Weight Changed, Stroke, Fatty Liver, Urate Lowering Therapy, NLARBs, and GCs.

MIMIC-IV：The basic model included UA,Age,CCB, Statins, Diuretic, Losartan, β-blocker, GCs, NLR, Urate Lowering Therapy, and eGFR.

Inpatient Gout Recurrence With Normal Serum Uric Acid

GoutRe：The basic model included UA, Tophus, Stroke, eGFR, Dyslipidemia1, NLR, Tophus, and GCs.

MIMIC-IV：The basic model included UA, Age, Diuretic, GCs, NLR, and Tophus.

Cumulative Incidence of Inpatient Gout RecurrenceAmong Patients Undergoing Urate−Lowering Therapy

GoutRe：The basic model included UA, Tophus, Drinking History, Dyslipidemia,Losartan, and GCs

MIMIC-IV：The basic model included Tophus, Diuretic, and GCs.

Supplementary Figures

eFigure 1. Overview of Study Design

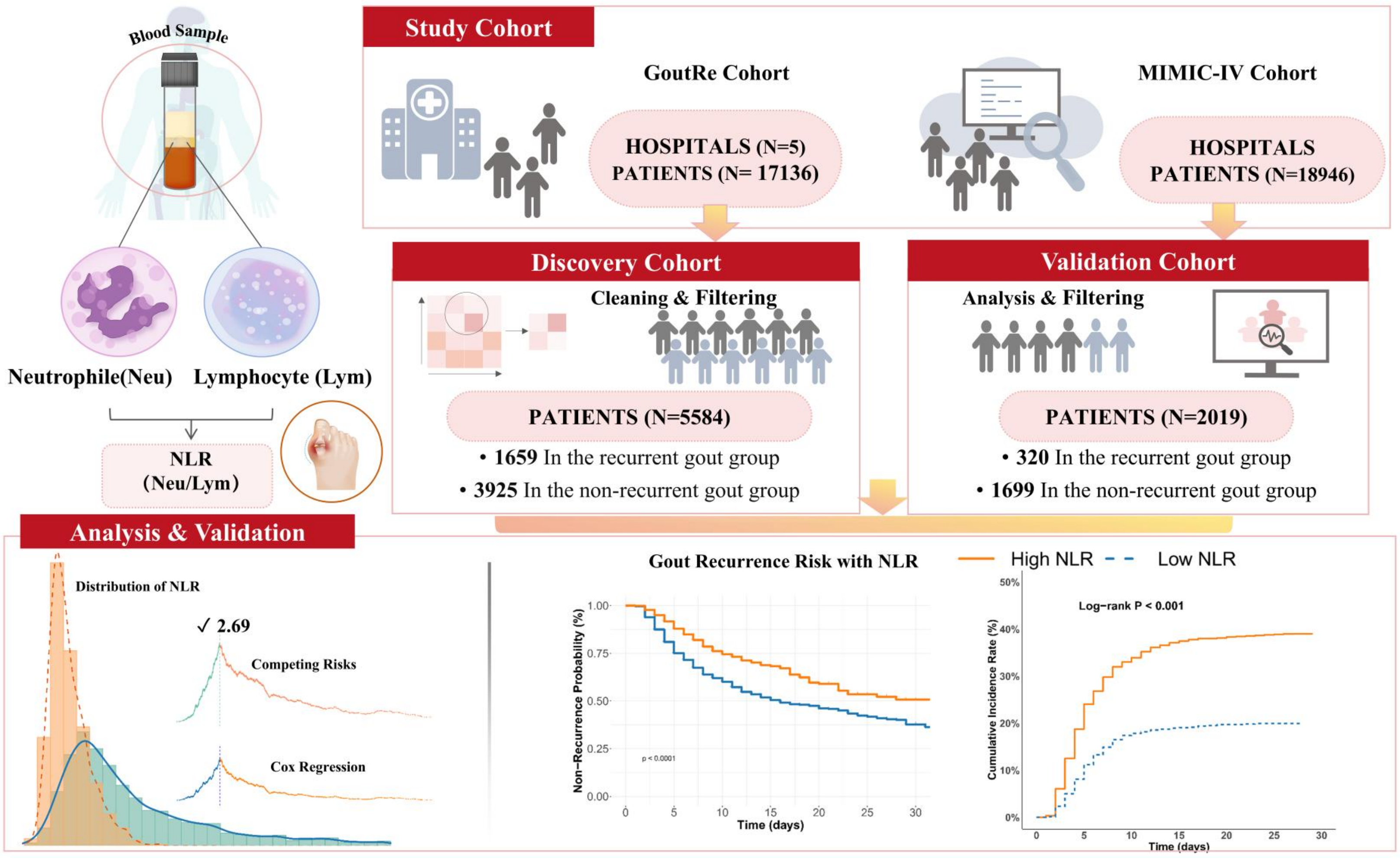

Abbreviations: NLR: Neutrophil-Lymphocyte Ratio.

eFigure 2. Correlation and ROC Curve Analyses Between NLR and CRP for Predicting Inpatient Gout Recurrence

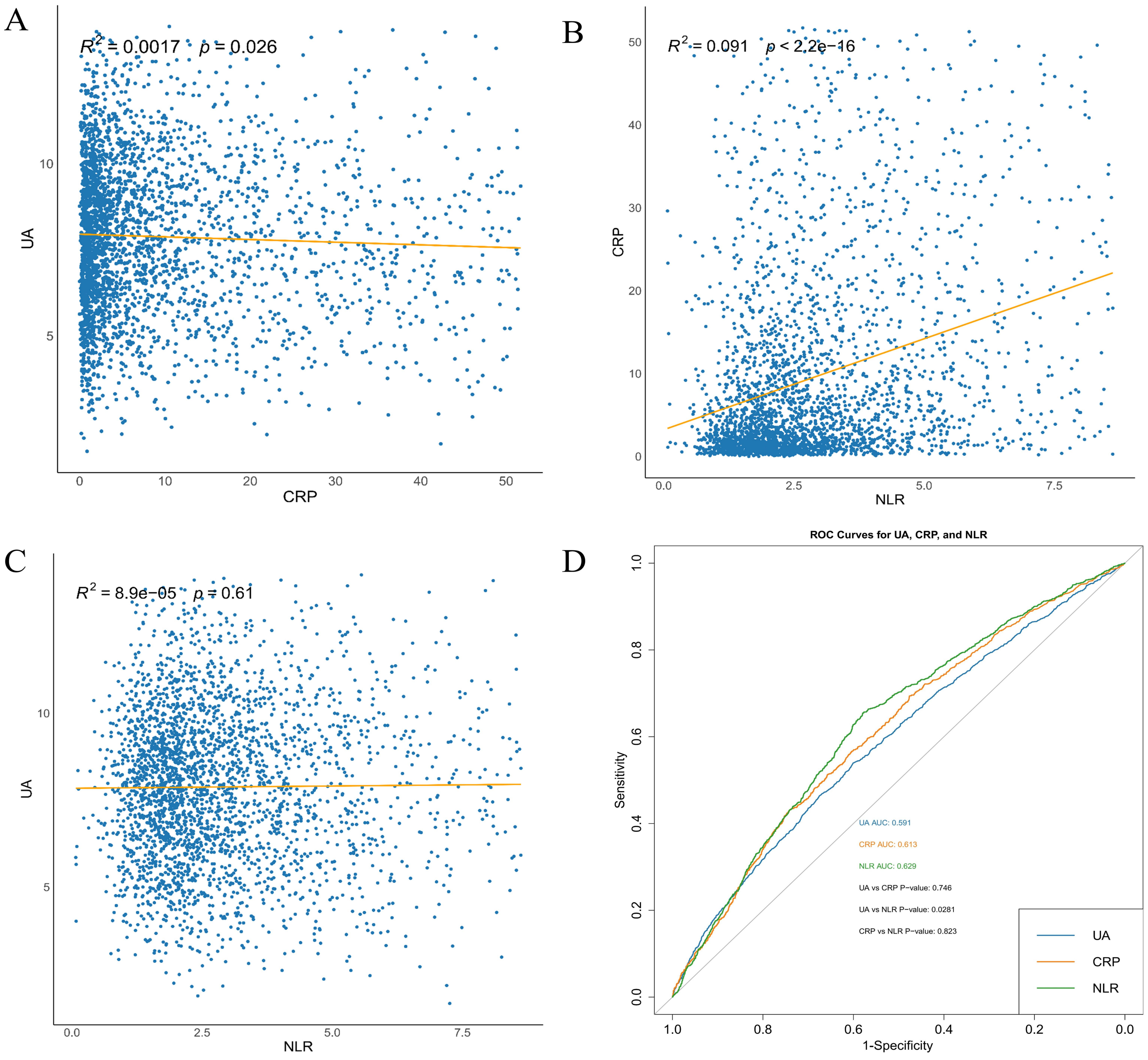

Abbreviations: NLR, Neutrophil-Lymphocyte Ratio, no units; CRP, C-reactive protein (mg/L); UA, Uric acid (mg/dL).

eFigure 3. Kaplan-Meier Survival Curve for Inpatient Gout Recurrence in Subgroup in the GoutRe Cohort and the MIMIC-IV Cohort

GoutRe

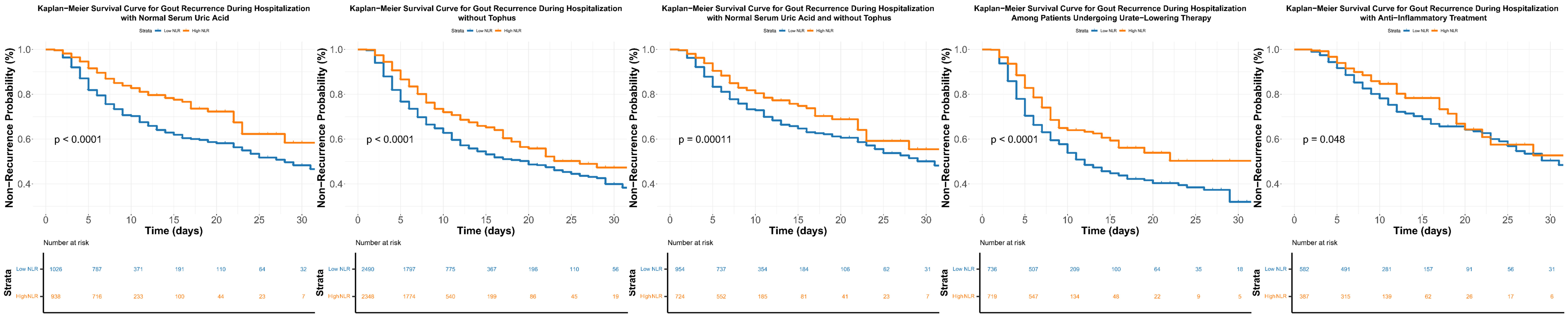

MIMIC-IV

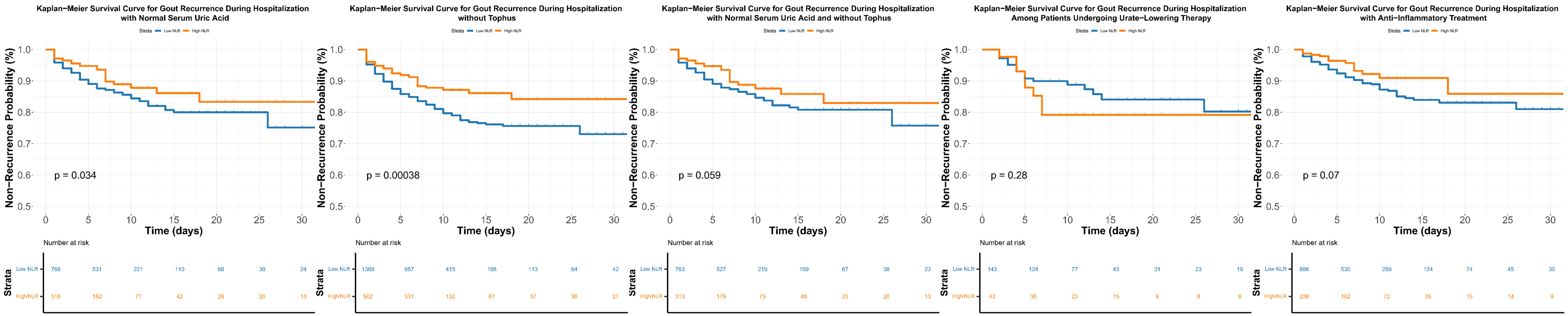

eFigure 4. Kaplan-Meier Survival Curve for Inpatient Gout Recurrence in Subgroup Under Contrasting Conditions in the GoutRe Cohort and the MIMIC-IV Cohort

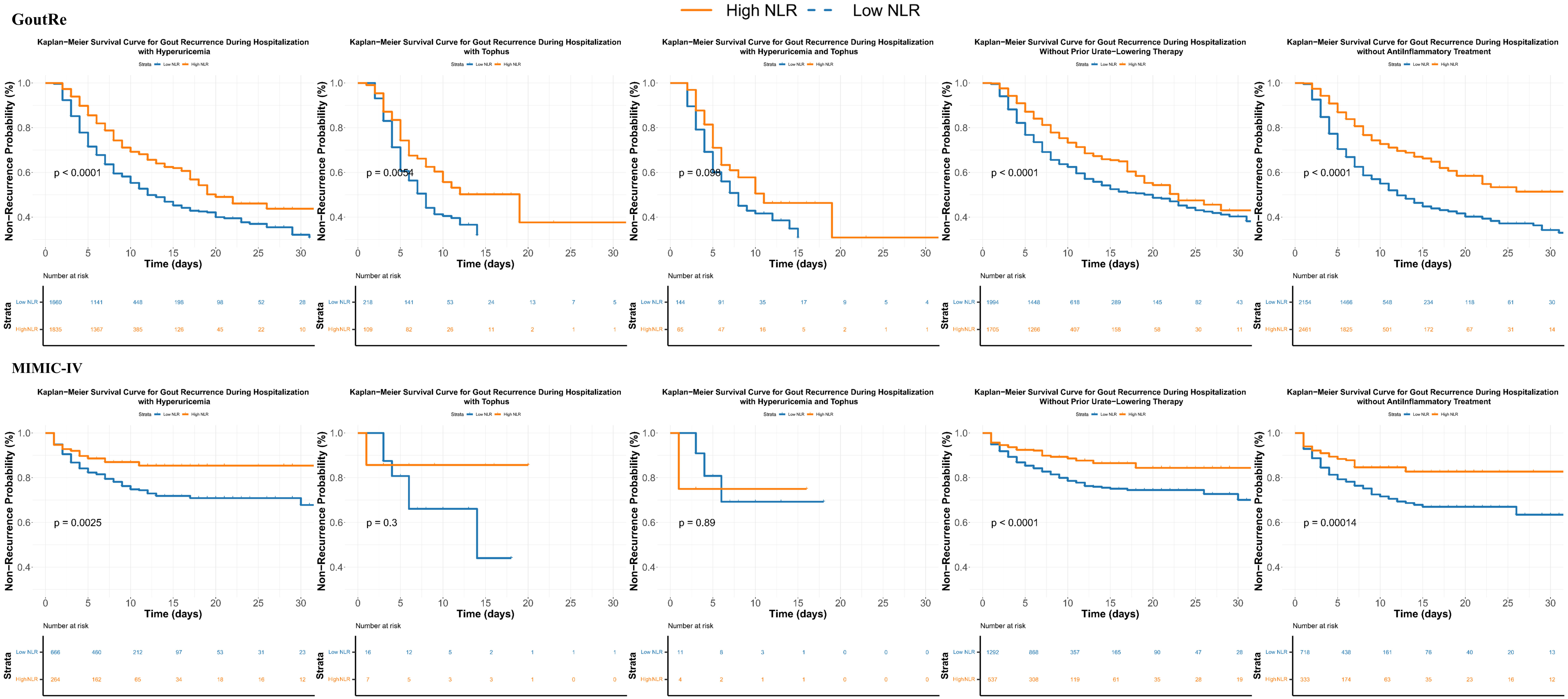

eFigure 5. Cumulative Incidence of Inpatient Gout Recurrence in Subgroup in the GoutRe Cohort and the MIMIC-IV Cohort

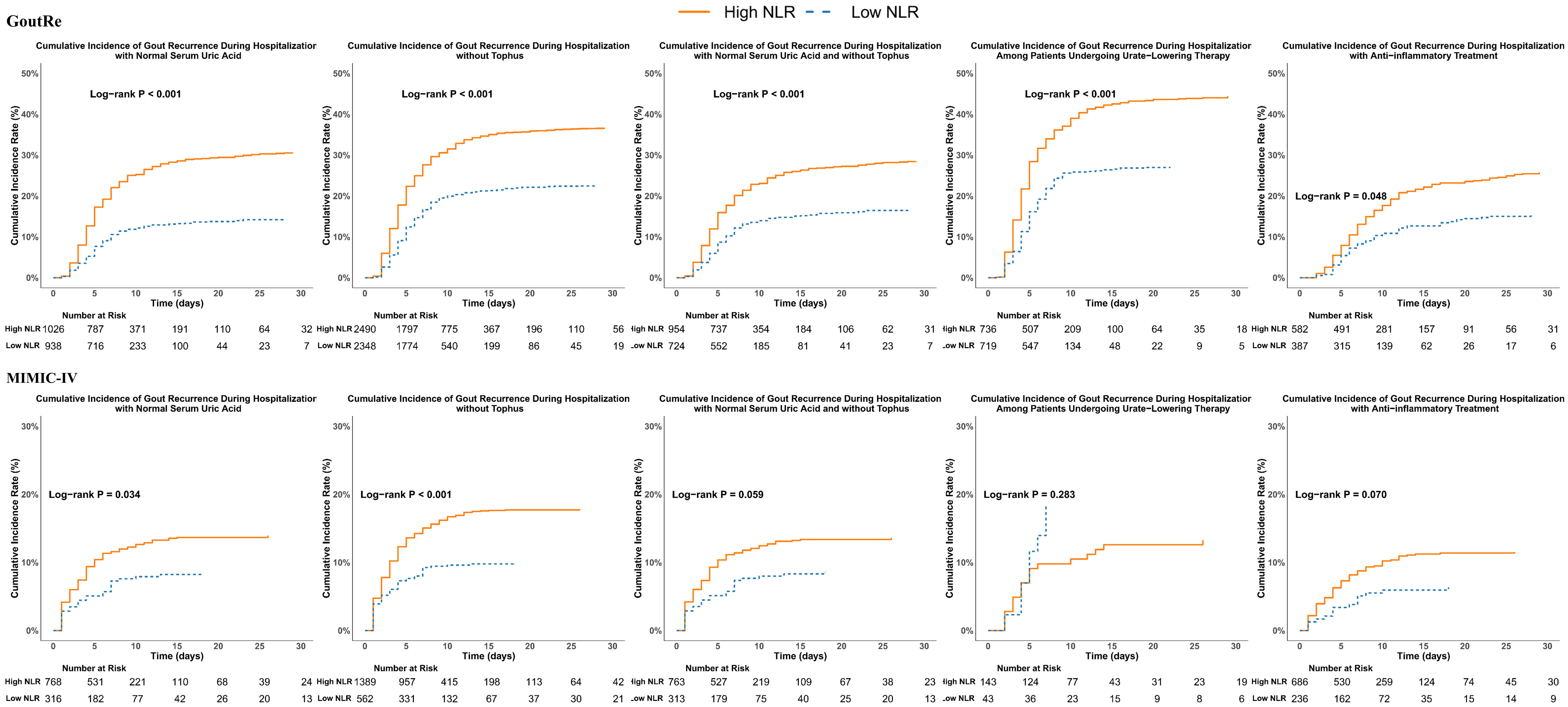

eFigure 6. Cumulative Incidence of Inpatient Gout Recurrence in Subgroup Under Contrasting Conditions in the GoutRe Cohort and the MIMIC-IV Cohort

GoutRe

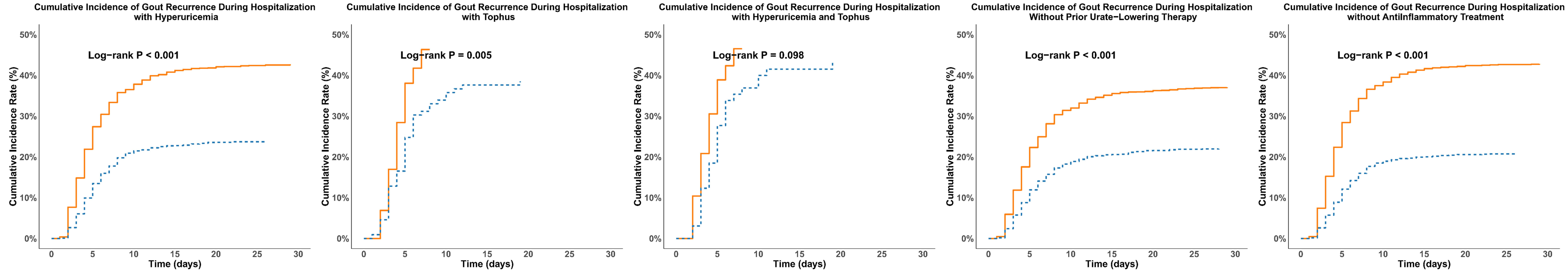

Number at Risk (with Hyperuricemia)

	0	5	10	15	20	25	30
High NLR	1660	1141	448	198	98	52	28
Low NLR	1835	1367	385	126	45	22	10

Number at Risk (with Tophus)

	0	5	10	15	20	25	30
High NLR	218	141	53	24	13	7	5
Low NLR	109	82	26	11	2	1	1

Number at Risk (with Hyperuricemia and Tophus)

	0	5	10	15	20	25	30
High NLR	144	91	35	17	9	5	4
Low NLR	65	47	16	5	2	1	1

Number at Risk (Without Prior Urate−Lowering Therapy)

	0	5	10	15	20	25	30
High NLR	1994	1448	618	289	145	82	43
Low NLR	1705	1266	407	158	58	30	11

Number at Risk (without AntiInflammatory Treatment)

	0	5	10	15	20	25	30
High NLR	2154	1466	548	234	118	61	30
Low NLR	2461	1825	501	172	67	31	14

MIMIC-IV

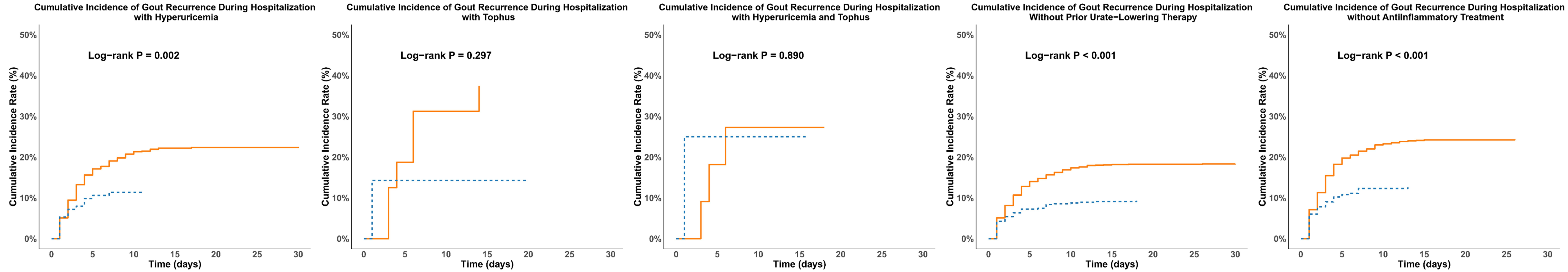

Number at Risk (with Hyperuricemia)

	0	5	10	15	20	25	30
High NLR	666	460	212	97	53	31	23
Low NLR	264	162	65	34	18	16	12

Number at Risk (with Tophus)

	0	5	10	15	20	25	30
High NLR	16	12	5	2	1	1	1
Low NLR	7	5	3	3	1	0	0

Number at Risk (with Hyperuricemia and Tophus)

	0	5	10	15	20	25	30
High NLR	11	8	3	1	0	0	0
Low NLR	4	2	1	1	0	0	0

Number at Risk (Without Prior Urate−Lowering Therapy)

	0	5	10	15	20	25	30
High NLR	1292	868	357	165	90	47	28
Low NLR	537	308	119	61	35	28	19

Number at Risk (without AntiInflammatory Treatment)

	0	5	10	15	20	25	30
High NLR	718	438	161	76	40	20	13
Low NLR	333	174	63	35	23	16	12

eFigure 7. Decision Curve Analysis for Inpatient Gout Recurrence Prediction Models in the GoutRe and MIMIC-IV Cohorts

GoutRe cohort

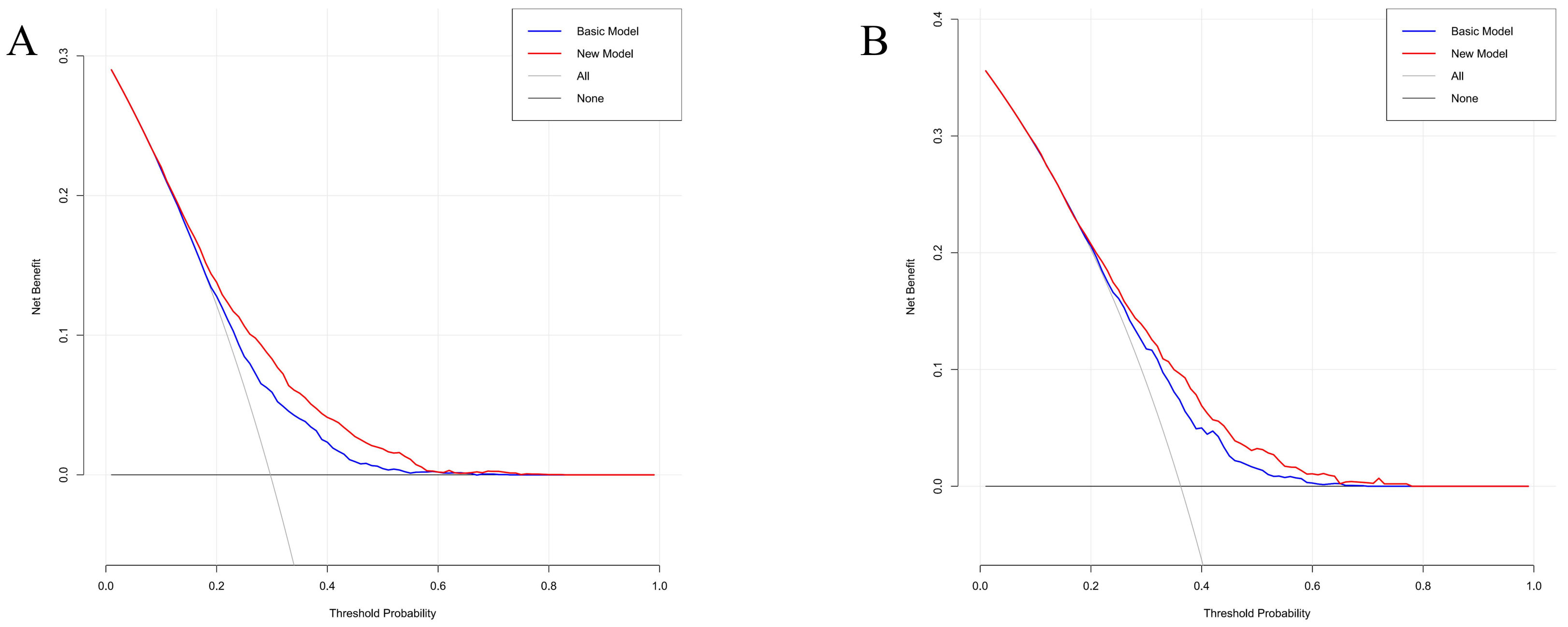

MIMIC-IV cohort

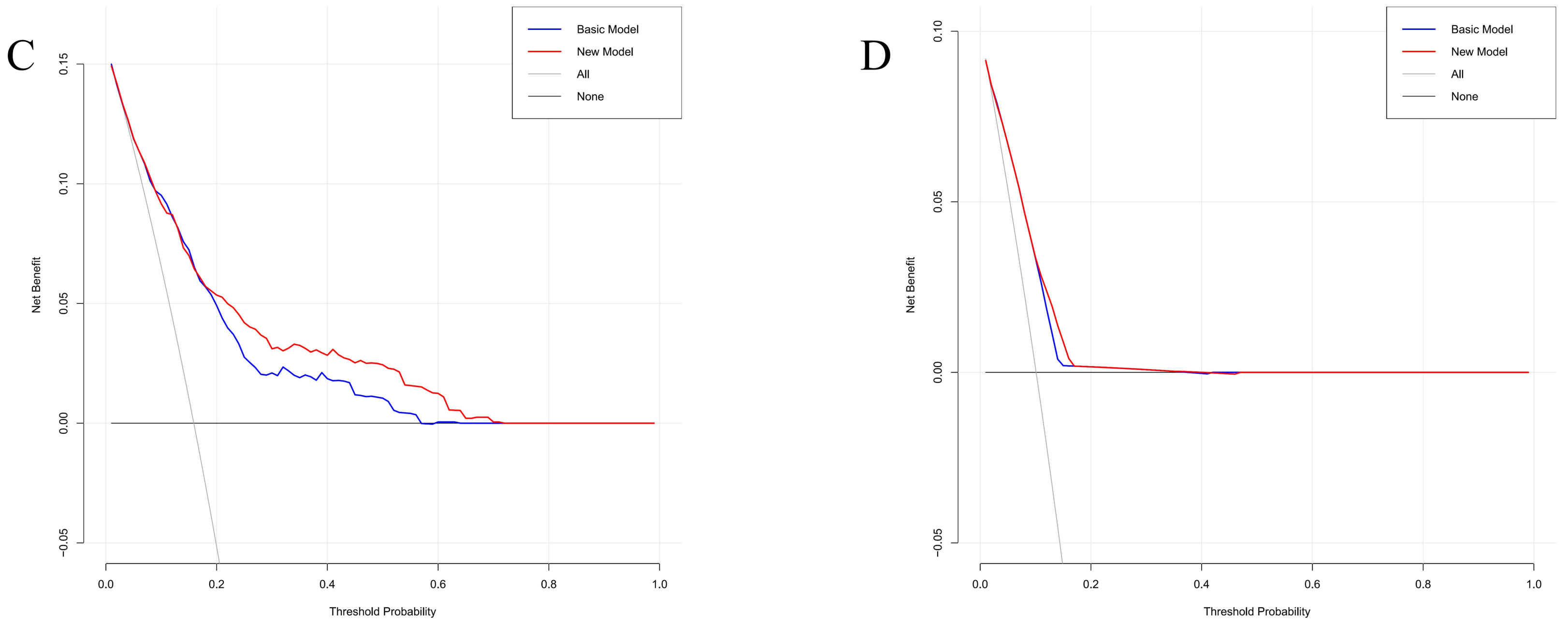

The decision curve analysis (DCA) compares the net benefit of two predictive models for inpatient gout recurrence across different threshold probabilities in both the GoutRe and MIMIC-IV cohorts. The blue line represents the basic model, while the red line represents the new model, which includes the neutrophil-to-lymphocyte ratio (NLR). The new model consistently demonstrates a higher net benefit across a wider range of threshold probabilities compared to the basic model in both cohorts（A, C）, particularly in patients undergoing urate-lowering therapy (B, D)

REFERENCES:

[1] Johnson A E W, Stone D J, Celi L A, et al. The MIMIC Code Repository: enabling reproducibility in critical care research[J]. Journal of the American Medical Informatics Association, 2018,25(1):32-39.

[2] Johnson A E W, Bulgarelli L, Shen L, et al. MIMIC-IV, a freely accessible electronic health record dataset[J]. Scientific Data, 2023,10(1):1.

[3] Implementation of the International Statistical Classification of Diseases and Related Health Problems, Tenth Revision (ICD-10)[J]. Epidemiological bulletin - Pan American Health Organization, 1997,18(1):1.

[4] World Health O. ICD-10 : international statistical classification of diseases and related health problems : tenth revision[Z]. 2nd ed ed. Geneva: World Health Organization, 2004.

[5] Stevens P E, Ahmed S B, Carrero J J, et al. KDIGO 2024 Clinical Practice Guideline for the Evaluation and Management of Chronic Kidney Disease[J]. Kidney international, 2024,105(4):S117-S314.

[6] World Health O. The use of stems in the selection of International Nonproprietary Names (INN) for pharmaceutical substances 2018 (StemBook2018)[R]. Geneva: World Health Organization, 2018.

[7] Major T J, Dalbeth N, Stahl E A, et al. An update on the genetics of hyperuricaemia and gout[J]. Nat Rev Rheumatol, 2018,14(6):341-353.

[8] Leung N, Yip K, Pillinger M H, et al. Lowering and Raising Serum Urate Levels: Off-Label Effects of Commonly Used Medications[J]. Mayo Clin Proc, 2022,97(7):1345-1362.
